# Identifying Multiomic Signatures of X‐Linked Retinoschisis‐Derived Retinal Organoids and Mice Harboring Patient‐Specific Mutation Using Spatiotemporal Single‐Cell Transcriptomics

**DOI:** 10.1002/advs.202405818

**Published:** 2024-11-06

**Authors:** Yueh Chien, You‐Ren Wu, Chih‐Ying Chen, Yi‐Ping Yang, Lo‐Jei Ching, Bo‐Xuan Wang, Wei‐Chao Chang, I‐Hsun Chiang, Pong Su, Shih‐Yu Chen, Wen‐Chang Lin, I‐Chieh Wang, Tai‐Chi Lin, Shih‐Jen Chen, Shih‐Hwa Chiou

**Affiliations:** ^1^ Department of Medical Research Taipei Veterans General Hospital Taipei 11217 Taiwan; ^2^ Institute of Pharmacology College of Medicine National Yang Ming Chiao Tung University Taipei 11221 Taiwan; ^3^ Institute of Food Safety and Health Risk Assessment National Yang Ming Chiao Tung University Taipei 11221 Taiwan; ^4^ Institute of Clinical Medicine School of Medicine National Yang Ming Chiao Tung University Taipei 11221 Taiwan; ^5^ Center for Molecular Medicine China Medical University Hospital Taichung 40447 Taiwan; ^6^ Institute of Biomedical Sciences Academia Sinica Taipei 11529 Taiwan; ^7^ Genome and Systems Biology Degree Program Academia Sinica and National Taiwan University Taipei 10617 Taiwan; ^8^ Institute of Biomedical Informatics National Yang Ming Chiao Tung University Taipei 11221 Taiwan; ^9^ School of Medicine College of Medicine National Yang Ming Chiao Tung University Taipei 11221 Taiwan; ^10^ Department of Ophthalmology Taipei Veterans General Hospital Taipei 112201 Taiwan; ^11^ Genomics Research Center Academia Sinica Taipei 11529 Taiwan

**Keywords:** chronic ER stress‐associated apoptosis, eIF2α signaling, genetically engineered mice, retinoschisin 1 (RS1), single‐cell RNA‐sequencing, spatiotemporal transcriptomics, X‐link retinoschisis (XLRS)

## Abstract

X‐linked retinoschisis (XLRS) is an inherited retinal disorder with severe retinoschisis and visual impairments. Multiomics approaches integrate single‐cell RNA‐sequencing (scRNA‐seq) and spatiotemporal transcriptomics (ST) offering potential for dissecting transcriptional networks and revealing cell‐cell interactions involved in biomolecular pathomechanisms. Herein, a multimodal approach is demonstrated combining high‐throughput scRNA‐seq and ST to elucidate XLRS‐specific transcriptomic signatures in two XLRS‐like models with retinal splitting phenotypes, including genetically engineered (*Rs1* ^emR209C^) mice and patient‐derived retinal organoids harboring the same patient‐specific p.R209C mutation. Through multiomics transcriptomic analysis, the endoplasmic reticulum (ER) stress/eukryotic initiation factor 2 (eIF2) signaling, mTOR pathway, and the regulation of eIF4 and p70S6K pathways are identified as chronically enriched and highly conserved disease pathways between two XLRS‐like models. Western blots and proteomics analysis validate the occurrence of unfolded protein responses, chronic eIF2α signaling activation, and chronic ER stress‐induced apoptosis. Furthermore, therapeutic targeting of the chronic ER stress/eIF2α pathway activation synergistically enhances the efficacy of AAV‐mediated *RS1* gene delivery, ultimately improving bipolar cell integrity, postsynaptic transmission, disorganized retinal architecture, and electrophysiological responses. Collectively, the complex transcriptomic signatures obtained from *Rs1* ^emR209C^ mice and patient‐derived retinal organoids using the multiomics approach provide opportunities to unravel potential therapeutic targets for incurable retinal diseases, such as XLRS.

## Graphical abstract

A multimodal approach is demonstrated integrating high‐throughput scRNA‐seq and ST to elucidate XLRS‐specific transcriptomic signatures in genetically engineered (*Rs1* ^emR209C^) mice and patient‐specific iPSC‐derived 3D‐retinal organoids harboring the patient‐specific p.R209C mutation. The most enriched disease pathway is identified and targeted therapeutically, showing synergistic efficacy when combined with AAV‐based Rs1 delivery in ameliorating XLRS phenotypes.

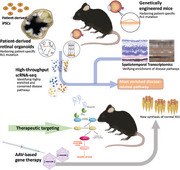

## 1 Introduction

X‐linked retinoschisis (X‐linked juvenile retinoschisis; XLRS) is an early‐onset X‐linked inherited retinal disorder that leads to structural splitting of the neurosensory retina and severe visual impairment.^[^ ^1^ ^]^ Retinoschisin‐1 (RS1) is a 224‐amino acid protein consisting of two conserved regions: an N‐terminal signal sequence (aa 1–23) and a discoidin domain (aa 64–219). Secreted by retinal cells, RS1 forms a homo‐octameric complex through disulfide bonds, maintaining retinal structural integrity by binding to photoreceptors and bipolar cells.^[^ ^2^ ^]^ The pathogenic mechanisms of XLRS have been extensively investigated by numerous elegant studies using *Rs1*‐knockout mice that perfectly recapitulated XLRS phenotypes.^[^ ^2^, ^3^ ^]^ Although AAV‐based *RS1* gene delivery has indeed shown promising efficacy in rescuing retinoschisis in *Rs1*‐knockout mice,^[^ ^2^, ^4^ ^]^ AAV‐based *RS1* gene therapy unexpectedly yielded unsatisfactory outcomes after an 18‐month treatment in a clinical trial.^[^ ^5^ ^]^ However, the exact factors contributed to the ineffective treatment of *RS1* gene therapy in XLRS patients remain unknown. To further optimize treatment strategies for XLRS, it is critically important to elucidate the precise pathogenic transcriptomic mechanisms.

Disease modeling is crucial for recapitulating phenotypes, studying pathogenesis, and conducting drug screening, thereby enhancing data reliability and clinical translation. In XLRS, over 190 mutations in the *Retinoschisin*‐1 (*RS1*) gene, primarily located within the discoidin domain,^[^ ^6^ ^]^ have been identified and are widely recognized as the primary cause of XLRS pathogenesis.^[^ ^7^ ^]^ Despite the valuable insights gained from XLRS research using *Rs1*‐knockout mice,^[^ ^2^, ^3^, ^4^ ^]^ this model does not incorporate the specific clinically relevant mutations found in patients. Mutations in the *RS1* gene can interfere with its octamer structure formation, leading to the production of misfolded RS1 protein. This misfolding impedes the protein's transport to the cell surface, significantly contributing to the pathologies associated with XLRS.^[^ ^8^ ^]^ In this context, some research groups have developed knock‐in models with patient‐specific *Rs1* mutations, providing crucial insights into how specific mutations contribute to pathological phenotypes in XLRS.^[^ ^9^ ^]^ For example, Chen et al. developed a *RS1‐*knock‐in mice harboring the causative mutation (*RS1*, p.Y65X) and these mice exhibited decreased b‐wave amplitude in the electroretinogram with the splitting of inner nuclear layer (INL), shortened inner segments and reduced outer segment numbers.^[^ ^9a^ ^]^ Liu et al. further generated knock‐in mice with different patient‐specific point mutations, i.e., C59S and R141C point mutants, and demonstrated that the early‐onset XLRS‐like disease phenotype is genotype‐dependent.^[^ ^9b^ ^]^ Regarding in vitro platforms modeling XLRS, patient‐induced pluripotent stem cell (iPSC)‐derived organoids also harbor patient‐specific mutations and hold promising potential for personalized therapy.^[^ ^10^ ^]^ Utilizing their 3D structure and functions, these organoids bridge the gap between in vitro studies and clinical applications.^[^ ^11^ ^]^ Patient iPSC‐derived organoids have been used to model neurodegeneration diseases^[^ ^12^ ^]^ and cystic fibrosis.^[^ ^13^ ^]^ Previously, we generated retinal organoids from XLRS patient iPSCs, and these patient‐derived retinal organoids replicate patient‐specific mutations and splitting phenotypes.^[^ ^10a^ ^]^ These observations highlighted the importance of the distinct contribution of specific point mutations to disease severity in XLRS.

Modern omics technologies have shown promising potential in identifying disease biomarkers and screening potential therapeutics, therefore exploiting their merits in biomedical or translational applications.^[^ ^14^ ^]^ Single‐cell RNA sequencing (scRNA‐seq) is a recently rising technology and powerful tool for investigating cellular heterogeneity and measuring gene expression at an individual cell level.^[^ ^15^ ^]^ The technique has been extensively used for biomarker identification,^[^ ^14^ ^]^ pathogenesis investigation,^[^ ^16^ ^]^ and disease diagnosis.^[^ ^17^ ^]^ Remarkably, scRNA‐seq has also been utilized in human retinas and retina organoids to examine the changes in transcriptomic landscape during eye development^[^ ^18^ ^]^ and the alterations of biological pathways in ocular diseases, including age‐related macular degeneration, autoimmune retinopathy, and retinoblastoma.^[^ ^19^ ^]^ More recently, the spatiotemporal transcriptomics (ST) technique has been developed utilizing in situ experiments to understand the gene expression and cell‐cell interaction spatially. Relying on such technologies, new subtypes of retinal cells have been identified based on their spatial arrangement.^[^ ^20^ ^]^ ScRNA‐seq and ST have been combined to decipher the molecular mechanisms during retinal development.^[^ ^21^ ^]^ However, these integrative transcriptomic approaches have not yet been utilized in retinal diseases. Therefore, a multimodal sequencing platform that integrates scRNA‐seq and ST may allow the spatial assessment of the differential gene expression between individual cells within the interested niche at single‐cell levels, further providing us the opportunities for exploring disease pathogenesis of incurable vision disorders, such as XLRS.

In this study, we proposed a multiomic approach integrating ST and scRNA‐seq to investigate the intricate transcriptomic signatures associated with XLRS. We employed CRISPR‐Cas9 technology to create genetically engineered mice carrying the patient‐specific p.R209 mutation (*Rs1* ^emR209C^). These *Rs1* ^emR209C^ mice exhibited typical XLRS‐like phenotypes, including retinal splitting and impaired electrophysiological function. Using the integrated multiomic approach, we analyzed the transcriptomic signatures of the *Rs1* ^emR209C^ mouse retina as well as the XLRS patient‐derived retinal organoids, as described in our previous work.^[^ ^10a^ ^]^ By identifying overlapping transcriptomic signatures between these two models, we pinpointed the most enriched disease‐related pathways and delineated potential pathogenic mechanisms. Through subsequent wet‐lab experiments and liquid chromatography‐mass spectrometry (LC/MS)‐based proteomic analysis, we validated the chronic activation and enrichment of identified disease‐related pathways. Notably, therapeutic targeting of the chronic activation of endoplasmic reticulum (ER) stress/eukaryotic initiation factor 2 (eIF2) signaling using salubrinal, a small molecule specific inhibitor that suppresses chronic ER stress‐induced apoptosis^[^ ^22^ ^]^ and alleviates ER workload,^[^ ^23^ ^]^ mitigated XLRS‐like phenotypes in *Rs1* ^emR209C^ retinas. Combining this targeted therapy with AAV‐based *RS1* gene delivery demonstrated synergistic efficacy, suggesting a novel and effective treatment strategy for the currently incurable XLRS.

## 2 Results

### 2.1 Modeling XLRS Using Patient‐Derived Retinal Organoids and Genetically Engineered Mice

**Figure 1:**
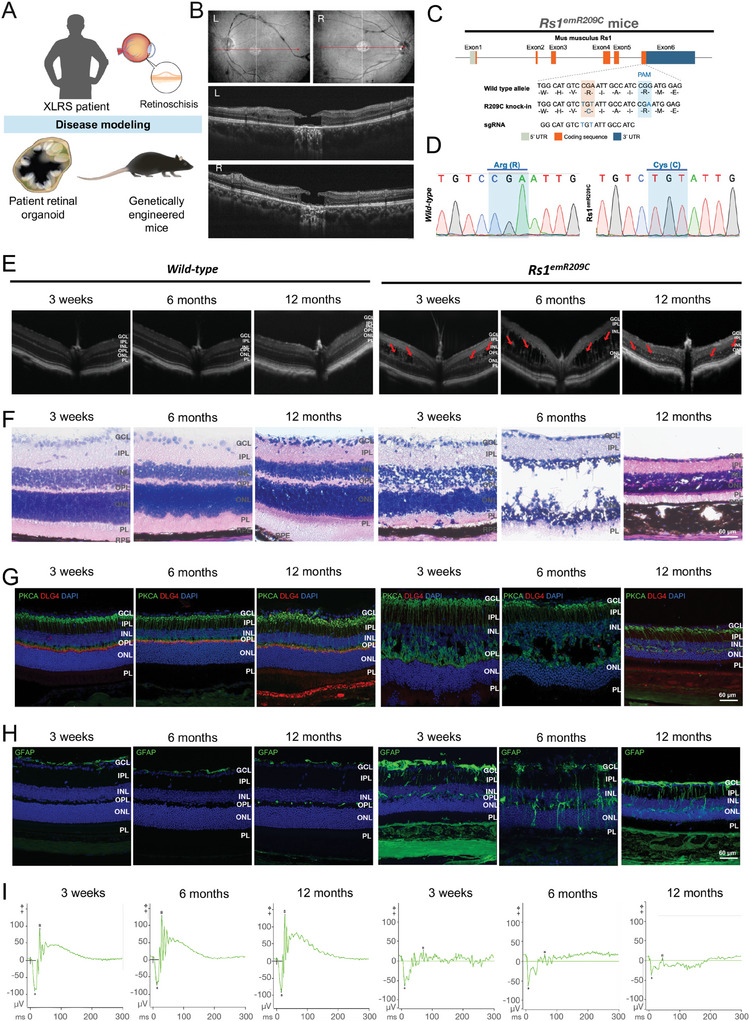
Characterizing XLRS patient‐derived retinal organoids and genetically engineered *Rs1* knock‐in mice carrying patient‐specific point mutation p. R209C. A) Schematic illustration showing the disease modeling using XLRS patient‐derived retinal organoids and genetically engineered *Rs1* knock‐in mice (*Rs1* ^emR209C^ mice). Note that the patient‐derived retinal organoids and *Rs1* ^emR209C^ mice carried the point mutation p. R209C identical to that of the enrolled XLRS patient. B) Clinical examination of XLRS patient with p. R209C point mutation by OCT imaging and color fundus photography. C) The design of the CRISPR/Cas9 genome editing system to generate *Rs1* ^emR209C^ mice by introducing sgRNA to target the PAM sequence on the wild‐type allele. D) Confirmation of introducing point mutation p. R209C into *Rs1* in *Rs1* ^emR209C^ mice using Sanger sequencing. E) OCT imaging and F) H & E staining of *Rs1* ^emR209C^ and age‐matched wild‐type mouse retinas at different ages. G) IF staining shows the expression of bipolar cell marker, PRKCA (green), and postsynaptic marker, DLG4 (red) in *Rs1* ^emR209C^ and age‐matched wild‐type mouse retinas at different ages. Nuclei were stained with DAPI (blue). H) IF staining and quantification of GFAP stained müller glia (green) and in *Rs1* ^emR209C^ and wild‐type retinas at different ages. I) Dark‐adapted ERG responses in *Rs1* ^emR209C^ and age‐matched wild‐type mouse retinas at different ages. Scale bar = 60 µm.

To establish XLRS‐like models, we generated in vivo genetically engineered mice harboring the specific point mutations found in XLRS patients and used in vitro patient iPSC‐derived retinal organoids carrying the same mutation, as described previously.^[^ ^10a^ ^]^ We then employed multiomic approaches to collect transcriptomic signatures from both models, aiming to elucidate the molecular pathogenic mechanisms of XLRS (**Figure** **1**A). With respect to the in vitro XLRS model, we enrolled an XLRS patient who carried the c.625C>T (p. R209C) mutation and exhibited typical XLRS features, as described previously.^[^ ^10a^ ^]^ The clinical findings of the ophthalmological survey and the patient's fundus photography showed macular atrophy and the characteristic “spoke‐wheel” appearance (Figure 1B; **left eye, upper left; right eye, upper right**). Optical coherence tomography (OCT) images revealed loss of the INL and outer nuclear layer (ONL) in the fovea and mild retinoschisis at the nasal site (Figure 1B; **left eye, middle; right eye, bottom**). We then collected peripheral blood mononuclear cells (PBMCs) from the XLRS patient and a healthy control donor to reprogram them into iPSCs and further differentiate them into retinal organoids. The bright‐field microscopy and hematoxylin and eosin (H&E) staining data confirmed that, in contrast to the retinal organoids from control subjects, the XLRS patient‐specific iPSC‐3D‐retinal organoids exhibited the XLRS‐specific splitting phenotype after 150 days of differentiation (Figure S1A,B, Supporting Information), consistent with our previous observations.^[^ ^10a^ ^]^ Sanger sequencing confirmed the specific *Rs1* point mutation and the alteration in amino acids of patient‐specific iPSCs, identical to that found in the respective patient (Figure S1C,D, Supporting Information). Next, we generated an XLRS knock‐in mouse model harboring the same patient‐specific p.R209C mutations via CRISPR/Cas9 technology (Figure 1C). Finally, mice with a knock‐in patient‐specific *RS1* mutation were generated, and Sanger sequencing was performed to confirm the change in the DNA codon CGA (arginine) to TGT (cysteine) in exon 6 of the *Rs1* gene (Figure 1D; Figure S1H, Supporting Information) in the knock‐in XLRS mouse model (abbreviated as *Rs1* ^emR209C^). The disorganization of retinal structures and impaired retinal electrophysiology were subsequently verified by OCT imaging and electroretinogram (ERG), respectively. The chronological changes in the structural integrity of the retinas of *Rs1* ^emR209C^ mice were evaluated via OCT imaging at 3 weeks, 6 months, and 12 months (Figure 1E). Remarkably, severe retinoschisis was initially observed in the INL at 3 weeks of age, and the size of the retinal splitting cavities increased at 6 months of age in *Rs1* ^emR209C^ mice (Figure 1E). To examine the lesion foci of retinoschisis in the ONL and INL of *Rs1* ^emR209C^ mice, we performed H&E staining at 3 weeks, 6 months, and 12 months in the wild‐type and *Rs1* ^emR209C^ mouse retinas (Figure 1F). Histological assessment revealed disorganization of the ONL and INL at 3 weeks of age and retinal splitting cavities at 6 months of age in *Rs1* ^emR209C^ mice (Figure 1F). At 12 months, the aforementioned schisis‐like structures were resolved, the outer plexiform layer (OPL) was diminished, and the ONL, INL, outer segment (OS), and inner segment (IS) were shortened in *Rs1* ^emR209C^ mouse retinas compared to those in wild‐type mouse retinas (Figure 1E,F). In contrast, there were no schisis‐like lesions or other significant changes in the wild‐type mice at any given age (Figure 1E,F).

We also conducted various experiments to characterize the disease features and eye fundus manifestations of *Rs1* ^emR209C^ mice at the molecular level. First, using immunofluorescence staining with DAPI to stain cell nuclei, we counted the cell numbers in the ONL of wild‐type and *Rs1* ^emR209C^ mouse retinas at different ages (Figure S1I,J, Supporting Information). The cell density in the ONL was lower in the *Rs1* ^emR209C^ mouse retinas at 3 weeks (58.4 vs 72.3 cells/1000 µm^2^), 6 months (44.6 vs 63.7 cells/1000 µm^2^) and 12 months (59 vs 69.4 cells/1000 µm^2^) than in the wild‐type retinas at the corresponding ages (Figure S1I,J, Supporting Information). Protein kinase C‐α (PKCA)‐positive bipolar cells and discs large homolog 4 (DLG4)‐labeled postsynapses in the OPL confirmed intact neurosensory structures in wild‐type retinas, while *Rs1* ^emR209C^ retinas exhibited reduced or absent DLG4 expression, indicating disrupted neurosensory networks in the OPL (Figure 1G). The reduction in photoreceptor numbers and disrupted OPL neurosensory networks were consistent with the findings in the XLRS mouse model described previously.^[^ ^2^, ^3^ ^]^ We also assessed the number of Müller glia in *Rs1* ^emR209C^ and control retinas by staining with the Müller glia marker, glial fibriliary acidic protein (GFAP). The number of GFAP^+^ Müller glia in the wild‐type mouse retinas was similar at all tested time points, and compared with those in the age‐matched control retinas, the area of Müller glia in the *Rs1* ^emR209C^ mouse retinas was higher (3 weeks: 2.6 vs 8.8%; 6 months: 1.8 vs 10.0%; 12 months: 2.2 vs 10.3%; Figure 1H; Figure S1K, Supporting Information). Furthermore, we assessed visual function in wild‐type and *Rs1* ^emR209C^ mice using ERG. The electrophysiological responses of the *Rs1* ^emR209C^ mice revealed a decrease in the amplitude of the scotopic a‐wave at 3 weeks, complete absence at 6 months and 12 months (Figure 1I; Figure S1L, Supporting Information), and the disappearance of dark‐adapted b‐wave responses at all time points compared to those of age‐matched controls (Figure 1I; Figure S1M, Supporting Information). In addition, the *Rs1* ^emR209C^ mice exhibited longer a‐wave implicit time at 12 months (Figure S1N, Supporting Information). Altered ERG responses in *Rs1* ^emR209C^ mice supported retinal anatomical abnormalities, highlighting the dysfunctional synaptic transmission between photoreceptors and bipolar cells in XLRS mice. Overall, the *Rs1* ^emR209C^ mice and patient‐specific iPSC‐derived 3D‐retinal organoids recapitulated the retinoschisis‐like phenotype of XLRS, and the multiple ophthalmic/eye fundus manifestations of *Rs1* ^emR209C^ mice presented the similar clinical features with disease progression in human XLRS patients.

### 2.2 ScRNA‐seq Profiling of Patient‐Derived Retinal Organoids and Retinas from Genetically Engineered *Rs1* ^emR209C^ Mice

**Figure 2:**
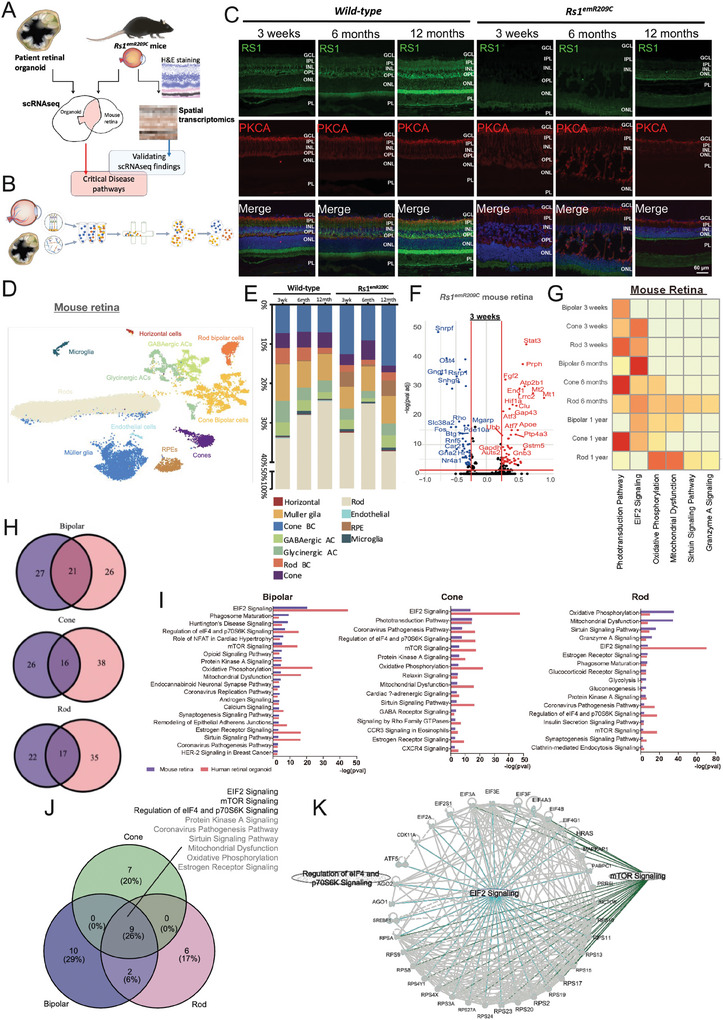
ScRNAseq analysis of *Rs1* ^emR209C^ mice and XLRS patient iPSC‐derived retinal organoids. A) Schematic illustration showing the multiomic approach integrating scRNA‐seq and ST to identify overlapping disease pathways on patient‐derived retinal organoids and *Rs1* ^emR209C^ mice. The transcriptomic signatures were identified by deep scRNA‐seq analysis from both models. The overlapping transcriptomic signatures identified by scRNA‐seq delineated the crucial disease‐related pathways. ST was used for validating the disease‐related pathways identified by scRNA‐seq. B)The diagram shows the scRNA‐seq experimental procedures of XLRS patient‐derived retinal organoids and *Rs1* ^emR209C^ mice. C) IF staining of RS1 (green) and bipolar cell marker PKCA (red) in *Rs1* ^emR209C^ and wild‐type retinas at different ages. Nuclei were stained with DAPI (blue). D) UMAP visualization and cell clustering of *Rs1* ^emR209C^ and age‐matched wild‐type mouse retinas. E) Relative proportions of cell clusters in *Rs1* ^emR209C^ and wild‐type retinas at different ages. F) Up‐regulated and down‐regulated DEGs of scRNA‐seq dataset in bipolar cells from *Rs1* ^emR209C^ and wild‐type mouse retinas at different ages. G) IPA results show the enriched pathways in the bipolar cells, cones, and rods of the Rs1^emR209C^ retinas at indicated times. H) Venn diagrams show the number of overlapped pathways enriched in bipolar cells, cones, and rods between *Rs1* ^emR209C^ retinas and XLRS patient‐specific retinal organoids. I) Overlapped pathways enriched in bipolar cells, cones, and rods of *Rs1* ^emR209C^ retinas and XLRS patient‐specific retinal organoids. J) Venn diagram shows the number of overlapped enriched pathways across bipolar cells, cones, and rods in *Rs1* ^emR209C^ retinas and XLRS patient‐specific retinal organoids. K) Gene interaction analysis reveals the interaction network among the genes involved in eIF2 signaling, mTOR and the regulation of the eIF4 and p70S6K signaling pathways.

To delineate critical disease‐related pathways involved in XLRS pathologies, we proposed a multimodal spatiotemporal platform that applies scRNA‐seq and ST on patient‐derived retinal organoids and *Rs1* ^emR209C^ mice (**Figure** **2**A). We collected both patient‐derived retinal organoids and *Rs1* ^emR209C^ mice and subjected them to high throughput scRNA‐seq, and identified critical disease‐related pathways in the overlap of the transcriptomic data from both models and subsequently validated the scRNA‐seq findings using ST (Figure 2A,B). Retinoschisis is predominantly observed in the INL of clinical XLRS patients^[^ ^24^ ^]^ and RS1 is expressed in bipolar cells and photoreceptors in the retina.^[^ ^25^ ^]^ Thus, we performed immunofluorescence staining to verify the expression and localization of the RS1 protein and the integrity of bipolar cells in wild‐type and *Rs1* ^emR209C^ retinas prior to scRNA‐seq. In wild‐type mice, the RS1 protein was predominantly localized to the IS, OPL, and INL, in which OPL and IPL were positively stained by the well‐known bipolar cell marker PKCA (Figure 2C). In contrast, the *Rs1* ^emR209C^ retina exhibited a significant reduction in RS1 expression in bipolar cells and photoreceptors, along with disorganization of PKCA‐expressing bipolar cells (Figure 2C). These data validated the localization of RS1 protein and its disease‐related expression pattern in *Rs1* ^emR209C^ mice. In scRNA‐seq, uniform manifold approximation and projection (UMAP) plots were generated for visualization. By analyzing retinas from *Rs1* ^emR209C^ and wild‐type mice, we identified 11 different clustered cell types, including rod bipolar cells, cone bipolar cells, cones, rods, and Müller glia, among a total of 37 342 cells and 21014 genes (Figure 2D; Figure S2A, **left**; Figure S2B, **upper;** Supporting Information). For the retinal organoids derived from patient and control iPSCs, 10 different cell types, including rods, cones, and bipolar cells, were classified among a total of 42 293 cells and 30 579 genes (Figure S2C,A, **right**; Figure S2B, **Lower;** Supporting Information). Leveraging the advantages of *Rs1* ^emR209C^ mouse retinas with naïve 3D tissue architecture and patient‐specific mutations, we delved into the clusters between *Rs1* ^emR209C^ retinas and wild‐type retinas at all tested time points to identify the vulnerable retinal cell types in XLRS (Figure 2E). Across all groups, the total cell count of each group was equivalent. In wild‐type retinas, rod cells displayed a modest increase after 3 weeks of age and remained stable from 6 months onward. Müller glia, on the other hand, remained relatively unaffected across all ages in wild‐type mice. With the R*s1* ^emR209C^ mice ages, there was a noticeable decreasing trend in the proportions of rod and cone cells, while the proportion of Müller glia gradually increased (Figure 2E). Consistent with the observations in Figure S1I,J (Supporting Information), the scRNA‐seq‐based approach also indicated a reduction in photoreceptors and an increase in Müller glia in *Rs1* ^emR209C^ retinas.

Next, we performed a deep scRNA‐seq analysis of bipolar cells and photoreceptors to dissect the XLRS pathologies and disease‐related genes in both *Rs1* ^emR209C^ mice and XLRS patient‐derived retinal organoids. Along with the clustering of specific retinal cell types using scRNA‐seq from *Rs1* ^emR209C^ mouse retinas and patient‐derived retinal organoids, we identified down‐ and up‐regulated differentially expressed genes (DEGs) (avg log_2_FC >0.25, p‐value adj <0.05) in the *Rs1* ^emR209C^ retina and the patient‐specific retinal organoid bipolar cells at different time points (Figure 2F; Figure S2D,E, Supporting Information). To elucidate the pathways influenced by the mutation of *Rs1* in both in vivo and in vitro models, we performed the Ingenuity Pathway Analysis (IPA) of the DEGs in bipolar cells and photoreceptors at different time points (Figure 2G; Figure S2F, Supporting Information). The IPA results revealed that ER stress‐related signaling pathways, including eIF2 signaling, the protein ubiquitination pathway, and the unfolded protein response pathway, were consistently enriched in the bipolar cells, cones, and rods from the *Rs1* ^emR209C^ retinas and XLRS patient‐specific retinal organoids at all tested time points (Figure 2G; Figure S2F, Supporting Information). To further identify the pathways that are crucial and highly conserved in bipolar cells and photoreceptors between the mouse model and patient‐derived organoids, we found several shared pathways between the two aforementioned experimental platforms in bipolar cells, rods, and cone cells (21 pathways in bipolar cells, 16 pathways in cones, 17 pathways in rods; Figure 2H). The overlapped pathways among in vivo and in vitro XLRS models of bipolar cells, and cone and rod cells were listed in Figure 2I, and further overlapping of listed pathways was conducted to identify the most vulnerable pathways to *Rs1* mutation across three cell types (Figure 2J). The results demonstrated that eIF2 signaling was the most enriched pathway in *Rs1*‐expressing cell types from the two aforementioned XLRS‐like models. In addition to eIF2 signaling, mTOR signaling, and the regulation of the eIF4 and p70S6K signaling pathways were also significantly enriched in *Rs1* ^emR209C^ retinas and patient‐derived retinal organoids (Figure 2J). Gene interaction analysis further revealed the close interaction network among the genes involved in eIF2 signaling, mTOR, and the regulation of the eIF4 and p70S6K signaling pathways (Figure 2K). Collectively, we combined the transcriptomic information from both models and found eIF2 signaling and other concomitant partners as highly enriched and conserved pathways in *Rs1*‐expressing cells from *Rs1* ^emR209C^ mouse retinas and XLRS patient iPSC‐derived 3D retinal organoids.

### 2.3 In Situ ST of *Rs1* ^emR209C^ Retinas Validated the Enrichment of Disease‐Related Pathways Identified by scRNA‐seq

**Figure 3:**
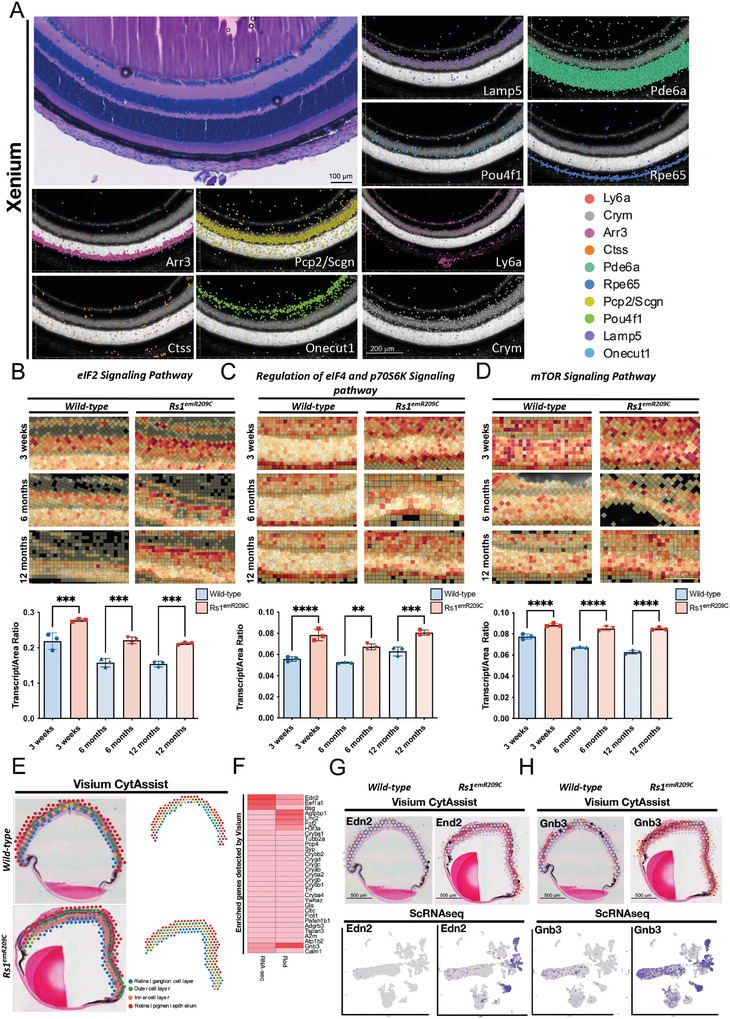
Chronic enrichment of ER stress/eIF2α pathway in RS1‐expressing cells in *Rs1* ^emR209C^ mouse retinas. A) Xenium analysis shows the visualization of selected marker genes to identify retinal ganglion cells, rod and cone photoreceptors, retinal pigment epithelium, microglia, and endothelial cells. The original H&E‐stained section is presented in the upper left corner. B) Xenium analysis shows the enrichment of (B) eIF2α pathway, C) regulation of eIF4 and p70S6K signaling pathway, and D) mTOR signaling pathway in *Rs1* ^emR209C^ retinas compared to age‐matched wild‐type retinas. Transcripts per area were quantified and shown as the lower subpanel in panels B‐D (N = 3, mean ± s.e.). E) Using CytAssist Visium to match the gene expression captured spots in the spatial transcriptome (right) in the H&E‐stained images of 3‐week‐old wild‐type and *Rs1* ^emR209C^ retinas (left). The captured spots were further divided into different retinal layers, including the retinal ganglion cell layer (blue), inner cell layer (orange), outer cell layer (green), and retinal pigment epithelium (red). F) Heatmap shows the expression of CytAssist Visium‐detected enriched genes in bulk RNAseq and the rod cell data from scRNA‐seq. CytAssist Visium‐captured spots shows the gene expression of (Panel G; Upper) *Edn2* and (Panel H; Upper) *Gnb3* in wild‐type and *Rs1* ^emR209C^ retinas. scRNA‐seq and UMAP visualization show the distribution of *Edn2* and *Gnb3* expression in cell clusters (Panels G, H; Lower).

Xenium, a novel in situ ST platform, is capable of capturing mRNA within cells and employs spatial labeling methods to project genetic data onto H&E‐stained or confocal images. Following the Xenium analysis, we first conducted marker gene analysis to visualize known cell markers and identify corresponding cells. We identified retinal ganglion cells, rod and cone photoreceptors, retinal pigment epithelium, microglia, and endothelial cells by staining for each specific marker, including *Ly6a, Crym, Arr3, Ctss, Pde6a, Rpe65, Pcp2/Scgn, Pou4f1, Lamp5*, and *Onecut1* (**Figure** **3**A; Upper left inset: H&E‐stained sections, the rest: Detection of gene expression using Xenium; Figure S3A,B, Supporting Information). Given that our findings identified eIF2 signaling as the most enriched disease‐related pathway in the overlapping transcriptomic signatures from *Rs1* ^emR209C^ retinas and patient‐derived retinal organoids, we then focused on analyzing the eIF2 signaling pathway after completing a layered analysis of the retinal cells (Figure 3B; Figure S3C, Supporting Information). In addition to assessing eIF2 signaling, we simultaneously assessed other enriched pathways, such as the mTOR pathway and the regulation of eIF4 and p70S6K pathways (Figure 3C,D), which were previously identified through scRNA‐seq in bipolar, rod, and cone cells (Figure 2). Consistent with our scRNA‐seq findings (Figure 2), the spatial mapping of selected genes indicated enrichment of eIF2 signaling, mTOR, and the regulation of eIF4 and p70S6K pathways at all given ages during the 1‐year observation in *Rs1* ^emR209C^ mouse retinas (Figure 3B–D).

In addition to Xenium, we applied another ST platform, Visium CytAssist, to spatially map gene expression in 3‐week‐old wild‐type and *Rs1* ^emR209C^ mouse retinas (Figure 3E). The location of the captured spots matched that of the H&E‐stained wild‐type and mutant retinas, and these spots were further classified into 4 retinal layers, namely, the retinal ganglion cell layer, the inner cell layer, the outer cell layer, and the retinal pigment epithelium (Figure 3E). The functional analysis of the shared DEGs in the ST, scRNA‐seq, and bulk RNA‐seq data indicated developmental abnormalities in the mutant retinas (Figure S3D, Supporting Information). In addition, among 34 enriched genes recognized in Visium CytAssist, *Gnb3* and *Edn2* were also up‐regulated in the bulk RNA‐seq and scRNA‐seq of rod cells (Figure 3F; Figure S3E, Supporting Information). *Gnb3* and *Edn2* are associated with the abnormalities in retinal phototransduction^[^ ^26^ ^]^ and gliosis,^[^ ^27^ ^]^ respectively. In both Visium CytAssist and scRNA‐seq, remarkable enrichment of *Edn2* and *Gnb3* was observed in the *Rs1^emR209C^* retinas compared to the wild‐type retinas (Figure 3G,H). As shown by scRNA‐seq, *Gnb3* and *Edn2* were particularly enriched predominantly in the rod cells and Müller glia of *Rs1* ^emR209C^ retinas (Figure 3G,H, **lower**). Collectively, our ST data provided spatiotemporal transcriptomic evidence that eIF2 signaling, the mTOR pathway, and the regulation of eIF4 and p70S6K pathways were all chronically enriched in XLRS pathogenesis, validating the crucial disease‐related pathways identified by scRNA‐seq.

### 2.4 Validation of the Enrichment of Endoplasmic Reticulum Stress/eIF2α Pathways in *Rs1* ^emR209C^ Retinas

ER stress is well recognized for triggering the activation of unfolded protein response (UPR) pathways. This process begins when glucose‐regulated proteins (GRPs) dissociate from three key ER transmembrane sensors: Inositol‐Requiring Protein 1 (IRE1), Activating Transcription Factor 6 (ATF6), and Protein Kinase R‐like ER Kinase (PERK). Within the UPR signaling cascades, PERK plays a crucial role by phosphorylating eIF2α, which subsequently initiates downstream signaling to inhibit protein synthesis. Meanwhile, IRE1 and ATF6 contribute to protein homeostasis by enhancing protein folding and degrading misfolded proteins, respectively.^[^ ^28^ ^]^ Under normal conditions, phosphorylated PERK‐induced eIF2α phosphorylation leads to a protective response by globally reducing protein production and selectively promoting the translation of Activating Transcription Factor 4 (ATF4).^[^ ^29^ ^]^ However, when ER stress persists, this adaptive UPR response is overwhelmed, causing phosphorylated PERK to switch to a pro‐apoptotic pathway by activating C/EBP homologous protein (CHOP), ultimately resulting in cell death.^[^ ^30^ ^]^ Along with the observations of the most enriched eIF2 pathways identified by scRNA‐seq and ST in *Rs1* ^emR209C^ retinas, we next validated the possible involvement of these critical disease‐related pathways and of other noxious cellular events in XLRS pathologies. We performed quantitative real‐time PCR (qRT‐PCR) to examine and compare the expression of indicated genes between *Rs1* ^emR209C^ retinas and wild‐type retinas. Overall, we verified the upregulation of genes associated with conserved ER stress pathway (*sXbp1* and *Atf4*) and ER stress target genes (*Hspa5*, *Ddit3*, *Dnajc3*, and *Grp94*) in *Rs1* ^emR209C^ retinas compared with wild‐type retinas (Figure S4A, Supporting Information). Meanwhile, we also observed the upregulation of apoptosis‐related genes (*Casp3* and *Fas*), oxidative stress‐related genes (*Sod1* and *Cat*), and autophagy‐related genes (*Lamp1* and *Lamp2*) in the *Rs1* ^emR209C^ retinas (Figure S4A, Supporting Information).

**Figure 4:**
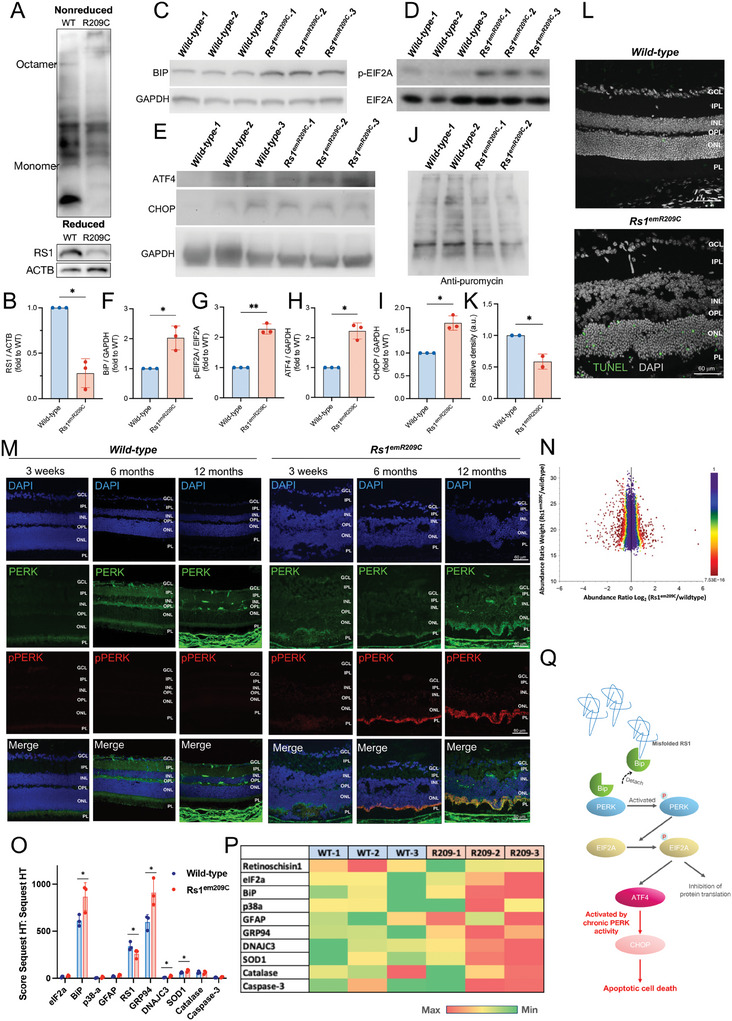
Misfolding of RS1 protein activates the ER stress/eIF2 pathway in *Rs1* ^emR209C^ mouse retinas. A) Nonreducing SDS polyacrylamide gradient gels showing the failure of RS1 homo‐octameric complex in *Rs1* ^emR209C^ mouse retinas (upper). In reducing gels, a large reduction of RS1 protein was observed in the lysates from *Rs1* ^emR209C^ mouse retinas (lower). B) Quantification of RS1 protein of wild‐type and *Rs1* ^emR209C^ mouse retinas in the reducing gel. Western blot shows increased C) BIP protein amount, D) the phosphorylation of eIF2α, E) ATF4 and CHOP protein amount in *Rs1* ^emR209C^ retinas compared to wild‐type retinas. Quantification of F) BIP protein amount, G) eIF2α phosphorylation, H) ATF4, and I) CHOP protein amount (N = 3, mean ± s.e.). (J) Puromycin incorporation assay indicated failure of protein synthesis in *Rs1* ^emR209C^ retinas (N = 2, mean ± s.e.). K) The quantification of protein synthesis through puromycin incorporation assay. L) TUNEL assay shows increased apoptosis in the *Rs1* ^emR209C^ retinas. M) IF staining shows increased phosphorylation of PERK in *Rs1* ^emR209C^ retinas at 6 months and 12 months, but no PERK phosphorylation was detected in wild‐type retinas at any given age. N) Mass spectrometry (LC/MS)‐based proteomic analysis showing the reduction in overall translation in the *Rs1* ^emR209C^ retinas (N = 4). O) Comparison of the score sequest HT:sequest HT between *wild‐typ*e and *Rs1* ^emR209C^ retinas at 3‐week ages (N = 3, mean ± s.e.). One‐way t‐test, *p‐value < 0.05. P) Heatmap comparing the expression of the candidate proteins involved in XLRS pathologies between *wild‐typ*e and *Rs1* ^emR209C^ retinas at 3‐week ages. Q) Schematic illustration shows RS1 protein misfolding chronically enriched ER stress and eIF2α pathway, impaired protein synthesis, leading to increased ER stress‐induced apoptosis in *Rs1* ^emR209C^ retinas.

Mutations in *RS1* can disrupt its octamer structure and produce misfolded RS1 protein, which hinders its transport to the cell surface.^[^ ^8^ ^]^ To verify whether the patient‐specific *RS1* mutation also induces RS1 misfolding in *Rs1* ^emR209C^ mouse retinas, we first examined whether the *Rs1* knock‐in R209C mutation affects the formation of the RS1 protein homo‐octameric complex. We analyzed the lysates from the retinas of both wild‐type and *Rs1* ^emR209C^ mice using nonreducing SDS polyacrylamide gradient gels (**Figure** **4**A, upper). As shown in the nonreduced gels, the wild‐type RS1 octamer migrated to an expected size of ≈180 kDa. In contrast, RS1 from *Rs1* ^emR209C^ mice failed to produce a mature complex despite the expression of the monomer (24 kDa; Figure 4A upper). In the reduced gels, total RS1 protein was largely decreased in mutant retinas compared to wild‐type retinas (Figure 4A lower, and 4B). These data indicated that the patient‐specific p.R209C mutation impaired protein folding and the formation of the homo‐octameric complex, consecutively contributing to reduced RS1 protein expression. Impaired protein folding and increased ER stress can induce the accumulation of BiP and the activation of UPRs.^[^ ^31^ ^]^ Among the three branches of UPR, the eIF2α pathway acutely reduced protein translation. Its downstream effector, ATF4, stimulates the expression of proteins involved in cell recovery and adaptation.^[^ ^30^ ^]^ Under prolonged ER stress, phosphorylated eIF2α further promotes the activation of ATF4 and CHOP, leading to ER stress‐induced apoptosis.^[^ ^28^, ^30^ ^]^ Accordingly, we next examined if the patient‐specific p.R209C mutation leads to prolonged ER stress in *Rs1* ^emR209C^ mice. Along with the observations of impaired RS1 protein folding, Western blot analysis revealed a robust increase in the protein levels of BiP (Figure 4C,F) and concomitant phosphorylation of eIF2α (Figure 4D,G). The protein levels of ATF4 and CHOP were also increased in *Rs1* ^emR209C^ retinas compared with wild‐type retinas (Figure 4E,H,I). These data indicated that long‐term failure of RS1 protein folding and assembly contributed to prolonged ER stress and upregulated eIF2α signaling. To examine whether this upregulation of eIF2α signaling affects protein translation, we used a protein synthesis assay to incorporate puromycin into nascent proteins and measure newly synthesized proteins as described previously.^[^ ^32^ ^]^ As detected by a puromycinylated protein antibody, newly synthesized proteins over a wide range of molecular weights were present in the control retinas. However, protein synthesis was barely detectable in the *Rs1* ^emR209C^ retinas (Figure 4J,K). Given that CHOP is the effector protein of ER stress‐induced apoptosis, we used a TUNEL assay to evaluate apoptosis in the *Rs1* ^emR209C^ retinas. Apoptotic signals were detected in the disorganized ONL in age‐matched *Rs1* ^emR209C^ retinas (Figure 4L). Furthermore, we used IF staining to identify the ER stress‐related proteins at the lesion sites in *Rs1* ^emR209C^ retinas and compared the expression of these proteins to wild‐type retinas. In the wild‐type retinas, no phosphorylated PERK (pPERK) was detected at any given age (Figure 4M, **left**). Compared to age‐matched wild‐type retinas, *Rs1* ^emR209C^ retinas showed no detectable PERK phosphorylation at 3 weeks of age but exhibited a time‐dependent increase in PERK phosphorylation in the IS and OS, where photoreceptors are located, at 6 months and 12 months (Figure 4M, **right**). Furthermore, we investigated the impact of the *Rs1* knock‐in p.R209C mutation on proteomic changes in *Rs1* ^emR209C^ retinas using (LC/MS)‐based proteomic analysis. At 3 weeks of age, *Rs1* ^emR209C^ retinas exhibited a significant reduction In overall protein expression compared to age‐matched wild‐type retinas (Figure 4N). The differences in protein expression between *Rs1* ^emR209C^ and wild‐type retinas were further analyzed. Notably, RS1 protein levels were significantly reduced in *Rs1* ^emR209C^ retinas, while ER molecular chaperones BiP and glucose‐related protein 94 (GRP94) were upregulated (Figure 4O,P). In addition to the findings of RS1, BiP, and GRP94, other enriched proteins included p38α (downstream target of the IRE1‐UPR branch), GFAP (the Müller glia marker), DNAJC3 (a chaperone of UPR), superoxide dismutase type 1 (SOD1) and catalase (antioxidant enzymes), and Caspase‐3 (a key mediator of apoptosis) (Figure 4O,P). These findings indicate the activation of the UPR and the impaired production of RS1 in *Rs1* ^emR209C^ retinas. Together, the *Rs1* ^emR209C^ mice exhibited XLRS phenotypes accompanied by failure of RS1 octamer structure formation, prolonged accumulation of ER stress and the enrichment of eIF2α pathway, impaired protein synthesis, and increased ER stress‐induced apoptosis (Figure 4Q).

### 2.5 Therapeutic Targeting of Chronic ER Stress/eIF2α Pathway Activation Ameliorated XLRS‐Associated Structural and Functional Abnormalities

**Figure 5:**
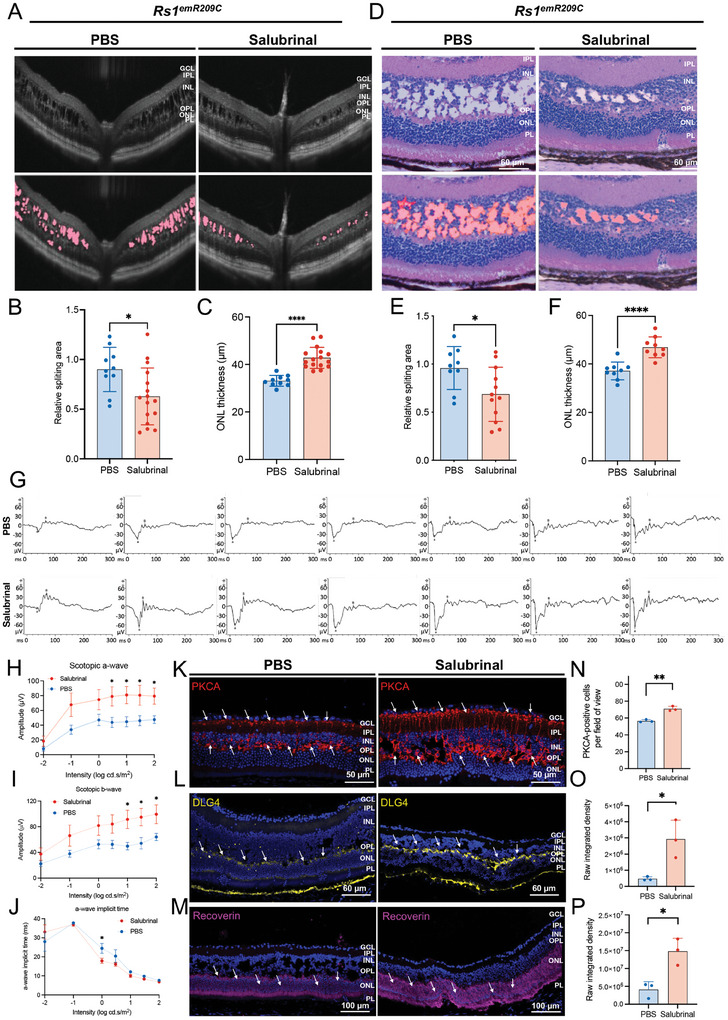
Therapeutic targeting the chronic ER stress/ eIF2 pathway activation ameliorates retinoschisis and improves retinal electrophysiological functions. A) OCT imaging of *Rs1* ^emR209C^ retinas with or without salubrinal treatment. B) Quantification of the schisis area based on the OCT imaging data of *Rs1* ^emR209C^ retinas with or without salubrinal treatment (N = 10, mean ± s.e.). C) Quantification of the ONL thickness based on the OCT imaging of *Rs1* ^emR209C^ retinas with or without salubrinal treatment (N = 10, mean ± s.e.). D) H & E staining of *Rs1* ^emR209C^ retinas with or without salubrinal treatment. E) Quantification of the schisis area based on the H&E staining of *Rs1* ^emR209C^ retinas with or without salubrinal treatment (N = 10, mean ± s.e.). F) Quantification of the ONL thickness based on the H&E staining data of *Rs1* ^emR209C^ retinas with or without salubrinal treatment (N = 10, mean ± s.e.). G) The intensity‐response relation for the dark‐adapted ERG of *Rs1* ^emR209C^ retinas with or without salubrinal treatment. H) The scotopic a‐waves, I) scotopic b‐waves, and J) the a‐wave implicit time of *Rs1* ^emR209C^ retinas with and without salubrinal treatment. In panels H, I, and J, the results are mean ± s.e. of eight independent experiments. K) IF staining of bipolar cell marker PKCA (red), L) postsynaptic marker DLG4 (yellow), and M) photoreceptor marker recoverin (purple) in *Rs1* ^emR209C^ retinas treated with salubrinal or PBS. Nuclei were stained with DAPI (blue). N) Quantification of the PKCA‐positive cells, and intensity of the area positive for O) DLG4, and P) recoverin in *Rs1* ^emR209C^ retinas treated with salubrinal or PBS. In panels N, O, and P, the results are mean ± s.e. of three independent experiments.

Salubrinal has been identified as a selective inhibitor of the GADD34/protein phosphatase 1 complex, which is responsible for the dephosphorylation of eIF2α.^[^ ^22^ ^]^ This small molecule exhibits potent efficacy in suppressing apoptosis induced by ER stress, while it does not affect apoptosis unrelated to ER stress.^[^ ^22^ ^]^ The cytoprotective effects of salubrinal are widely attributed to its ability to prolong eIF2α phosphorylation, which in turn inhibits global protein translation, reduces the ER's workload, and downregulates ER stress markers.^[^ ^23^ ^]^ Furthermore, some studies also reported that salubrinal might also influence the IRE1/p38‐dependent pathway,^[^ ^33^ ^]^ a distinct UPR branch, leading to a reduction in downstream apoptotic cell death. This effect might be associated with the overall decrease in ER stress linked to prolonged eIF2α phosphorylation, though this remains speculative and is not yet fully understood.^[^ ^23^ ^]^ To test the contribution of dysregulated ER stress and the chronically enriched eIF2α pathway to the XLRS‐like phenotypes in *Rs1* ^emR209C^ retinas, we chronically treated mutant mice with salubrinal to inhibit eIF2α phosphatase, which led to constitutive activation of eIF2 pathway signaling and the mitigation of ER unfolded protein load in *Rs1* ^emR209C^ mice. Either salubrinal (1 mg kg day^−1^) or PBS was intraperitoneally administered to 3‐week‐old *Rs1* ^emR209C^ mice for 28 consecutive days, after which the treated mice were subjected to phenotypic assessments (Figure S5A, Supporting Information). After a 28‐day salubrinal treatment, OCT images showed the reduced area of retinal splitting cavities (**Figure** **5**A,B) and increased ONL thickness (Figure 5C) in the salubrinal‐treated *Rs1* ^emR209C^ retinas compared to the PBS‐treated *Rs1* ^emR209C^ retinas. To verify the treatment efficacy of salubrinal, we further performed H&E staining to assess retinoschisis lesions in the ONL and INL retinal layers of *Rs1* ^emR209C^ retinas treated with either PBS or salubrinal. After salubrinal treatment, the stained images revealed a significant reduction in the number of retinal splitting cavities in the retinas of salubrinal‐treated *Rs1* ^emR209C^ mice compared with the retinas of the PBS‐treated *Rs1* ^emR209C^ mice (Figure 5D,E). An increase in ONL thickness was also observed in *Rs1* ^emR209C^ retinas via H&E staining (Figure 5F). Next, we recorded the dark‐adapted ERG responses to light stimuli at various intensities to evaluate the electrophysiological function of the *Rs1* ^emR209C^ retinas (Figure 5G). Compared to the PBS‐treated *Rs1* ^emR209C^ mouse, the light response curves of scotopic a‐wave and b‐wave were upward‐shifted by the administration of salubrinal (Figure 5H,I). The maximal a‐wave amplitudes in the salubrinal‐treated retinas were achieved under light stimulation at 1 log cd.s m^−2^ (salubrinal‐treated retinas vs PBS‐treated retinas = 80.86 vs 44.70 µV; Figure 5G,H). The b‐wave amplitude elicited by light stimulation at 1 log cd.s m^−2^ was 91.41 µV (salubrinal‐treated retinas vs PBS‐treated retinas = 91.41 vs 49.71 µV; Figure 5G,I). The a‐wave implicit time was significantly shortened by light stimulation at 0 log cd.s m^−2^ in the salubrinal‐treated retinas than in the PBS‐treated retinas of *Rs1* ^emR209C^ mice (salubrinal‐treated retinas vs PBS‐treated retinas = 17.88 ms vs 24.44 ms; Figure 5G,J). In addition to the OCT imaging and electroretinogram findings, we employed immunofluorescence to evaluate the efficacy of salubrinal on the integrity of the neurosensory network in the disorganized retinas of *Rs1* ^emR209C^ mice. Compared to PBS‐treated *Rs1* ^emR209C^ retinas, salubrinal treatment significantly increased the presence of PKCA‐stained bipolar cells in the inner plexiform layer (IPL), INL, and OPL layers (Figure 5K,N). Similarly, DLG4‐labeled structures were notably enhanced in the OPL layers following salubrinal treatment (Figure 5L,O). These results indicate that salubrinal rescued bipolar cells and restored the postsynaptic density of neurons in the OPL. Moreover, the enrichment of recoverin in the ONL layer suggested that salubrinal treatment mitigated photoreceptor loss in *Rs1* ^emR209C^ retinas (Figure 5M,P). Collectively, these immunofluorescence findings demonstrate that salubrinal improved bipolar cell integrity, photoreceptor survival, and postsynaptic transmission from photoreceptors to bipolar cells. This data highlights the potential of targeting ER stress‐associated apoptosis to ameliorate the severity and progression of XLRS in *Rs1* ^emR209C^ retinas, supporting the role of chronic eIF2 pathway activation in the disruption of retinal architecture, progressive retinoschisis, and impaired visual function in the XLRS mouse model.

### 2.6 In Situ ST Evidence Verifies the Diminishment of Disease‐Related pathways in Salubrinal‐Treated *Rs1* ^emR209C^ Mice

**Figure 6:**
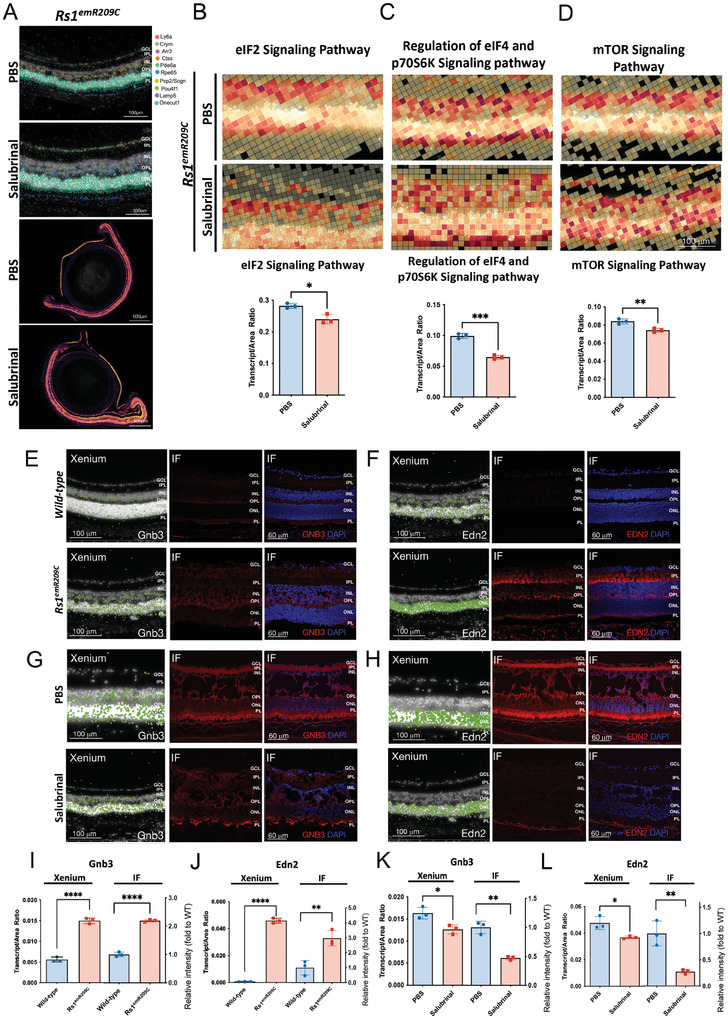
In situ spatial transcriptomics of differential gene expression in *Rs1* ^emR209C^ mouse retinas. A) Using the Xenium ST to visualize several cell types in *Rs1* ^emR209C^ mouse retina receiving the administration of salubrinal or PBS. Xenium analysis and quantification of the transcripts per area of the Xenium data showing the treatment effect of salubrinal on the enrichment of B) eIF2α pathway, regulation of C) eIF4 and p70S6K signaling pathway, and D) mTOR signaling pathway in *Rs1* ^emR209C^ retinas compared to PBS treated retinas (N = 3, mean ± s.e.). Xenium analysis and IF show the enrichment of E) Gnb3 and F) Edn2 at transcriptomic (left) and protein levels (middle and right) in *Rs1* ^emR209C^ retinas compared to age‐matched wild‐type retinas. Xenium analysis and IF show the effect of salubrinal treatment on the enrichment of G) Gnb3 and H) Edn2 gene at transcriptomic (left) and protein levels (middle and right) in *Rs1* ^emR209C^ retinas compared to PBS‐treated retina. The quantification of I) Gnb3 and J) Edn2 expression based on the Xenium analysis and IF data for Rs1^emR209C^ retinas and wild‐type retinas (N = 3, mean ± s.e.). The quantification of K) Gnb3 and L) Edn2 expression based on the Xenium analysis and IF data for Rs1^emR209C^ retinas treated with salubrinal or PBS (N = 3, mean ± s.e.).

To examine the post‐treatment outcome on the crucial disease pathways identified by the multimodal transcriptomic approach, we additionally performed Xenium ST to assess the spatial expression profiles of crucial disease pathways in *Rs1* ^emR209C^ mice after therapeutic targeting. Using marker gene analysis, the cell markers were visualized to identify the corresponding retinal cell types in *Rs1* ^emR209C^ mouse retina receiving the administration of salubrinal or PBS. Indicated retinal cell types were shown via staining for specific markers (**Figure** **6**A). Along with the observations in Figures 2, 3, 4, 5, we focused on the spatiotemporal mapping of DEGs corresponding to eIF2 signaling, mTOR, and the regulation of eIF4 and p70S6K pathways (Figure 6B–D). Among the retina samples from 3‐week‐old *Rs1* ^emR209C^ mice treated with salubrinal or PBS, we distinguished and analyzed the genes related to eIF2 signaling pathway, mTOR pathway, and the regulation of eIF4 and p70S6K pathways and quantified the signals to compare the differential expression of these genes between treated and untreated mice (Figure 6B–D). Compared to PBS treatment, salubrinal treatment effectively suppressed the enrichment of the genes related to these pathways in the ONL layer. Meanwhile, we also verified the changes in *Gnb3* and *Edn2* at the transcript and protein levels using Xenium analysis and immunofluorescence (Figure 6E–H). Consistent with the findings of Visium CytAssist and scRNA‐seq, the Xenium results also revealed the upregulation of *Gnb3* and *Edn2*, which were predominantly localized at the ONL of *Rs1* ^emR209C^ retinas at 3 weeks of age (Figure 6E,F,I,J). The protein levels of Gnb3 and Edn2 were also increased in the ONL layer (Figure 6E,F,I,J). We also evaluated and compared the expression of *Gnb3* and *Edn2* in *Rs1* ^emR209C^ retinas treated with salubrinal or PBS (Figure 6G,H,K,L). Xenium and IF data indicated that salubrinal treatment effectively abrogated the upregulation of *Gnb3* and *Edn2* at both the transcriptional and protein levels **(right in** Figure 6G,H,K,L). The diminishment of *Gnb3* and *Edn2* by salubrinal treatment suggested the alleviation of retinal gliosis and abnormal phototransduction. Overall, these data provided spatiotemporal transcriptomic evidence revealing the therapeutic outcome on the crucial disease pathways identified by the multimodal transcriptomic platforms.

### 2.7 Therapeutic Targeting of Chronic ER Stress/eIF2α Pathway Activation Enhanced the Efficacy of AAV‐Mediated *RS1* Gene Delivery

**Figure 7:**
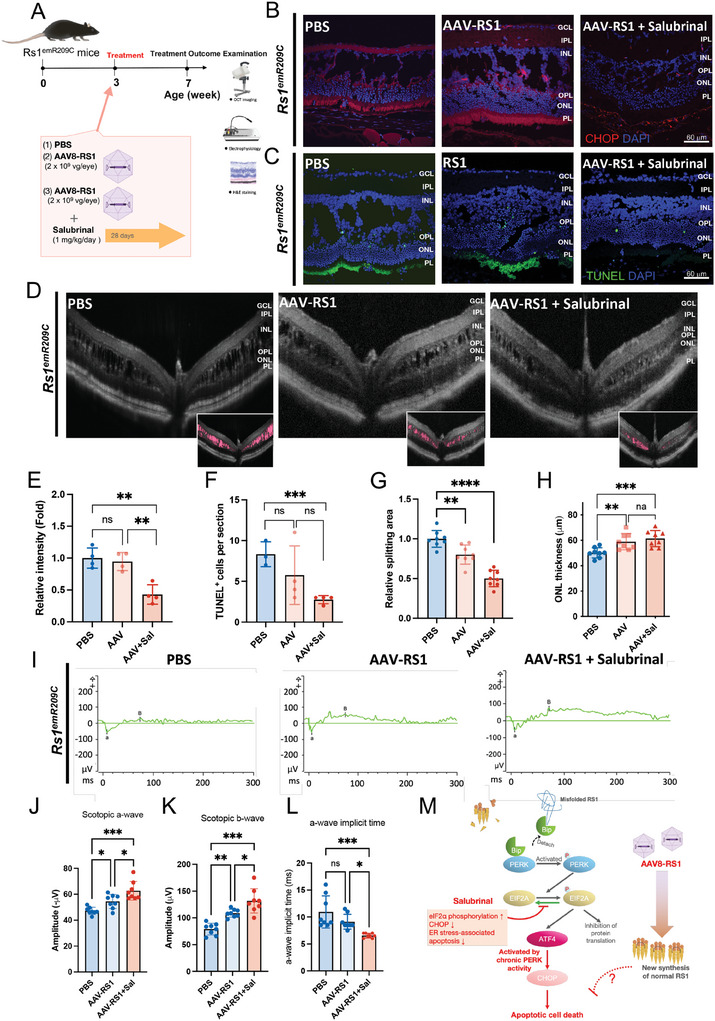
Combination of salubrinal administration and AAV‐based RS1 gene delivery synergistically ameliorates retinoschisis and improves retinal electrophysiological functions in *Rs1* ^emR209C^ mouse retinas. A) The experimental design shows the combination of salubrinal administration and AAV‐based RS1 gene delivery in *Rs1* ^emR209C^ retinas. B) CHOP staining and C) TUNEL assay in PBS‐treated *Rs1* ^emR209C^ retinas, AAV8‐based RS1 gene delivery, and the combination of salubrinal and AAV8‐based RS1 delivery. D) OCT imaging of *Rs1* ^emR209C^ PBS‐treated retinas, AAV8‐based RS1 gene delivery, and the combination of salubrinal and AAV8‐based RS1 delivery. The schisis splitting area (pink) is presented in the lower right corner. The quantification of E) CHOP protein content (N = 4, mean ± s.e.), F) apoptotic signals (N = 4, mean ± s.e.), G) the area of schisis splitting cavities (N = 7, mean ± s.e.), and H) ONL thickness (N = 8, mean ± s.e.) in *Rs1* ^emR209C^ retinas with indicated treatment are shown. I) The intensity‐response relation for the dark‐adapted full‐field ERG of PBS‐treated *Rs1* ^emR209C^ mouse retinas, AAV8‐based RS1 gene delivery, and the combination of salubrinal and AAV8‐based RS1 delivery. J) Comparison of the scotopic a‐wave amplitudes, K) scotopic b‐wave amplitudes, and L) the a‐wave implicit time of the PBS‐treated *Rs1* ^emR209C^ retinas, AAV8‐based RS1 gene delivery, and the combination of salubrinal and AAV8‐based RS1 delivery (N = 8, mean ± s.e.). M) A schematic diagram shows the enrichment of ER stress/eIF2 signaling pathways in XLRS pathogenesis. Misfolded RS1 protein increased ER stress, BIP, phosphorylated eIF2α, ATF4, and CHOP. This signaling cascade leads to the increase of apoptosis of retinal cells. Salubrinal that ameliorates ER stress and attenuates the eIF2 pathway exhibited a synergistic efficacy with AAV‐based RS1 gene delivery in the treatment of XLRS.

Several studies have reported the impressive efficacy of AAV‐based *RS1* gene delivery in XLRS mouse models.^[^ ^4^, ^34^ ^]^ These studies primarily utilized *Rs1*‐knockout mice, and the AAV‐based *RS1* gene delivery typically exhibited excellent efficacy after ≈4 to 6 months.^[^ ^2^, ^4^, ^35^ ^]^ In a subsequent clinical trial, AAV‐mediated *RS1* gene delivery generally produced unsatisfactory outcomes, only transiently closing retinoschisis cavities in one out of nine participants after 18 months.^[^ ^5^ ^]^ The exact mechanisms behind the inconsistent efficacy of *RS1* gene delivery remain unclear, likely due to some auxiliary disease mechanisms stemming from *RS1* gene mutations. To investigate whether the therapeutic targeting of the chronic eIF2α signaling activation could enhance the efficacy of AAV‐based *RS1* gene delivery, we intravitreally injected the human *RS1* gene through the AAV delivery system (2 × 10^9^ vg/eye) into 3‐week‐old *Rs1* ^emR209C^ mice and intraperitoneally injected either salubrinal (1 mg kg day^−1^) or PBS for 28 consecutive days. Phenotypic examination was also conducted after the 28‐day course of the experiment (**Figure** **7**A). Considering that CHOP is an effector of the eIF2 signaling pathway that contributes to ER stress‐induced apoptosis, we used IF and TUNEL assays to compare CHOP expression and changes in apoptotic signals in *Rs1* ^emR209C^ retinas with indicated treatments, respectively. AAV8‐mediated *RS1* gene delivery did not modify CHOP expression, whereas the combination of AAV8‐mediated gene delivery plus salubrinal modestly attenuated CHOP expression (Figure 7B,E). TUNEL‐positive apoptotic signals were consistently observed throughout the disorganized ONL in *Rs1* ^emR209C^ retinas. AAV8‐*RS1* treatment for 28 days showed a trend in reducing apoptotic cells but did not reach statistical significance (Figure 7C,F). Notably, salubrinal treatment plus AAV8‐*RS1* gene delivery effectively prevented the generation of apoptotic signals (Figure 7C,F).

**Figure 8:**
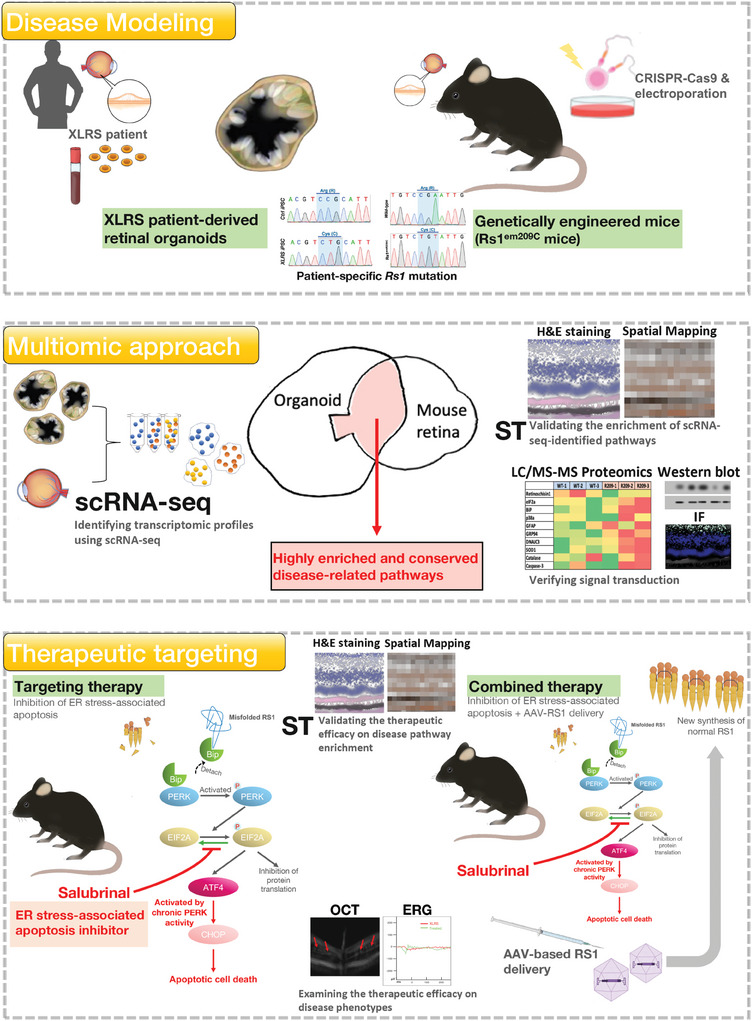
Schematic illustration showing the use of multiomic approaches to identify XLRS‐related disease pathways in in vitro and in vivo XLRS‐like models. For disease modeling (Upper), patient's peripheral blood was collected for the generation of patient iPSCs and patient iPSC‐derived retinal organoids. These patient‐derived retinal organoids carried patient's genetic background and unique splitting features. Meanwhile, using a CRISPR‐Cas9‐based technologies, we also generated the genetically engineered mice (*Rs1* ^emR209C^ mice) that exhibited XLRS‐like retinoschisis phenotypes. Sanger sequencing confirmed that both XLRS‐like models harbored the same patient‐specific *Rs1* point mutation. For the multiomic approach (Middle), we subjected the dissociated single cells from both patient‐derived retinal organoids and *Rs1* ^emR209C^ mouse retina to scRNA‐seq. The transcriptomic signatures of both XLRS‐like models identified by scRNA‐seq were integrated and overlapped to obtain the highly enriched and conserved pathways between two XLRS‐like models. In situ spatial transcriptomics was used to validate the enrichment of identified disease‐related pathways. The signal transduction of the disease‐related pathways were verified using immunofluorescence and Western blot. For the therapeutic targeting of the most enriched disease‐related pathway, a pharmacological agent was administered to target the most enriched disease pathway in the *Rs1* ^emR209C^ mice. The efficacy of therapeutic targeting on the enrichment of disease pathways were verified using in situ spatial transcriptomics. Its efficacy on disease phenotypes were examined using OCT and ERG. Therapeutic targeting plus AAV8‐mediated *Rs1* gene delivery further showed synergistic efficacy in *Rs1* ^emR209C^ mice. The therapeutic targeting inactivates the disease pathways and the gene therapy provided new wildtype RS1 protein.

As detected by OCT imaging, *Rs1* ^emR209C^ mice consistently exhibited severe retinal splitting at 3 weeks of age (Figure 7D). Compared to those in retinas from PBS‐treated *Rs1* ^emR209C^ mice, AAV8‐based *RS1* gene delivery led to a moderate reduction in retinal cavities, and the administration of salubrinal additionally decreased the cavity areas (Figure 7D,G). Similarly, an increase in ONL thickness was observed in AAV8‐*RS1*‐treated mice combined with salubrinal administration (Figure 7D,H). Subsequently, we assessed dark‐adapted ERG responses to light stimuli at various intensities in *Rs1* ^emR209C^ mice subjected to the indicated treatments (Figure 7I–L). Compared to those of *Rs1* ^emR209C^ mice injected with PBS, the light response curves of the scotopic a‐wave and b‐wave were mildly increased by AAV8‐*RS1* but largely increased by the combination of AAV8‐*RS1* and salubrinal administration (a‐wave: PBS‐treated retinas vs AAV8‐*RS1*‐treated retinas = 47.24 vs 54.47 µV; b‐wave: PBS‐treated retinas vs AAV8‐*RS1*‐treated retinas = 78.97 vs 108.71 µV; Figure 7I‐K). The b‐wave amplitude elicited by light stimulation at 1 log cd.s m^−2^ was 131.8 µV in the AAV8‐*RS1‐*salubrinal‐treated retinas. The combined treatment of AAV8‐*RS1* plus salubrinal exhibited a synergistic response in the elevation of b‐wave amplitude (PBS‐treated retinas vs AAV8‐*RS1‐*salubrinal‐treated retinas = 78.97 vs 131.8 µV; Figure 7I,K). The maximal a‐wave amplitude of the retinas was achieved under light stimulation at 1 log cd.s m^−2^ and the combination of AAV8‐*RS1* plus salubrinal also elicited the synergistic responses (PBS‐treated retinas vs AAV8‐*RS1*‐salubrinal‐treated retinas = 47.24 µV vs 62.675 µV; Figure 7I,J). Collectively, AAV8‐*RS1* moderately increased the amplitudes of the scotopic a‐ and b‐waves, while the addition of salubrinal synergistically enhanced the efficacy of AAV8‐*RS1* (Figure 7I–K). The a‐wave implicit time after light stimulation at 0 log cd.s m^−2^ was significantly shorter in the AAV8‐*RS1*‐salubrinal‐treated retinas than in the retinas of *Rs1* ^emR209C^ mice administered the other two treatments (PBS‐treated retinas vs AAV8‐*RS1*‐treated retinas vs AAV8‐*RS1*‐salubrinal‐treated retinas = 10.93 vs 9.125 vs 6.56 ms; Figure 7L). Overall, therapeutic targeting of the crucial disease pathways enhanced the efficacy of AAV‐mediated *RS1* gene delivery (Figure 7M). Taken together, our data have provided evidence that the chronic activation of ER stress/eIF2α signaling are crucial pathways for XLRS pathogenesis and progression. We demonstrated that multiomic approaches integrating ST and scRNA‐seq can effectively explore the complex transcriptomic signatures and pathomechanisms of XLRS both in vitro and in vivo. This platform also offers a personalized medicine‐based strategy for developing novel therapeutic targets to treat incurable ophthalmic disorders (**Figure** **8**).

## 3 Discussion

Advanced scRNA‐seq and ST allow precise exploration of disease‐specific transcriptomic signatures by analyzing differentially expressed genes at the single‐cell level and within their spatial context.^[^ ^14^, ^20^ ^]^ The eye consists of numerous small, intricate components that work together to focus on objects and transmit sensory information to the brain. Notably, the combination of scRNA‐seq and ST was employed to elucidate the transcriptomic mechanisms underlying retinal development.^[^ ^21^ ^]^ In the present study, we employed a multimodal approach combining ST and scRNA‐seq and integrated transcriptomic data from *Rs1^emR209C^* mouse retinas and patient iPSC‐derived retinal organoids, both harboring the same patient‐specific RS1 point mutations p.R209C. Using this multimodal spatiotemporal platform, we demonstrated that eIF2α signaling, the most enriched and highly conserved pathway, was chronically activated during disease progression in both in vivo and in vitro models (Figures 2 and 3). Through Western blots and LC/MS‐based proteomics, we found that this chronic eIF2α signaling activation is accompanied with the failure of RS1 protein folding, impaired protein synthesis, and ER stress‐induced apoptosis (Figure 4). Salubrinal, a small molecule inhibitor that targets the GADD34/protein phosphatase 1 complex to alleviate ER's workload and specifically inhibit ER stress‐induced apoptosis,^[^ ^22^, ^23^ ^]^ was used to therapeutically target the chronic activation of eIF2α signaling, demonstrating remarkable efficacy in mitigating retinoschsis and impaired electroretinogram responses (Figure 5). ST validated the restoration of chronically enriched pathways after salubrinal treatment (Figure 6). Importantly, salubrinal treatment improved the integrity of bipolar cells across the IPL, INL, and OPL layers, increased postsynaptic neuron density in the OPL, and attenuated photoreceptor loss in the OPL layers in *Rs1^emR209C^* retinas (Figure 5). The combination of AAV‐based *Rs1* delivery and salubrinal demonstrated synergistic efficacy in alleviating XLRS‐like phenotypes within 1 month.

The UPR is triggered by ER stress and initiates when GRPs detach from three key ER transmembrane sensors: IRE1, ATF6, and PERK. In the UPR pathways, PERK plays a critical role by phosphorylating eIF2α.^[^ ^28^ ^]^ Under normal conditions, this phosphorylation triggers a protective response by broadly reducing overall protein synthesis.^[^ ^29^ ^]^ However, when ER stress becomes prolonged or severe, phosphorylated PERK can no longer restore proteostatic balance and shifts toward a pro‐apoptotic pathway by activating the pro‐apoptotic transcription factor CHOP, ultimately leading to apoptotic cell death.^[^ ^30^ ^]^ Chronic ER stress has been implicated in various neurodegenerative diseases, including Alzheimer's^[^ ^36^ ^]^ and Parkinson's disease.^[^ ^37^ ^]^ However, as an early‐onset retinal degenerative disease, the role of chronic ER stress in XLRS has not yet been reported. Using non‐reducing gels, we observed the formation of misfolded RS1 protein in *Rs1^emR209C^* retinas. Western blot and LC/MS proteomics analysis revealed an enrichment of GRPs, such as BiP and GRP94 (Figure 4). The accumulation of GRPs is widely recognized as a trigger for UPR pathways, including the PERK/eIF2α branch. In *Rs1^emR209C^* retinas, the prolonged accumulation of misfolded RS1 protein may impair the eIF2α pathway's ability to maintain proteostasis, leading to CHOP activation and ER stress‐associated apoptosis. Our findings of enriched CHOP protein levels and increased TUNEL+ cells in *Rs1^emR209C^* retinas (Figure 4) support this interpretation: the chronic accumulation of misfolded RS1 proteins contributes to excessive ER stress, ultimately activating apoptotic cell death mechanisms. Salubrinal treatment that can ease ER stress, restore the proteostatic balance, and mitigate ER stress‐induced apoptosis significantly alleviated the severity of XLRS‐like phenotypes and improved sensory transmission in *Rs1^emR209C^* retinas (Figure 5). The findings of targeting study supported that chronic ER stress accumulation, the prolonged activation of eIF2α signaling, and ER stress‐associated apoptosis serve a critical role in the disruption of retinal architecture, progressive retinoschisis, and impaired visual function in *Rs1^emR209C^* retinas. Other highly enriched pathways identified by our multiomic approach included the mTOR pathway and the eIF4 and p70S6K pathways, both of which respond to ER stress.^[^ ^38^ ^]^ *Gnb3* and *Edn2*, two other enriched factors, were associated with impaired phototransduction^[^ ^26^ ^]^ and gliosis,^[^ ^27^ ^]^ respectively. Additionally, ST also showed that salubrinal suppressed the aforementioned enriched pathways, and the expression of *Gnb3* and *Edn2* in *Rs1* ^emR209C^ retinas. It is worthwhile to investigate the chronic activation of ER stress/eIF2 signaling in regulating the expression of *Gnb3* and *Edn2*, as well as their downstream interactions in progressive ocular disorders and severe retinal degeneration.

Although inconsistent outcomes in clinical trials of *RS1* gene delivery have been attributed to potential systemic immunogenicity against AAV and vector‐induced ocular inflammation,^[^ ^5^ ^]^ the exact mechanisms underlying the treatment's ineffectiveness in XLRS patients remain unclear. It is worth noting that in a single‐center, consecutive, retrospective study, 132 participants with confirmed XLRS, followed between 1999 and 2020, were found to harbor 66 *RS1* variants, 7 of which were novel, and exhibited diverse disease phenotypes.^[^ ^7^ ^]^ In vivo studies using knock‐in mice with various patient‐specific mutations have demonstrated that genotype is a key determinant of XLRS‐like phenotype severity.^[^ ^9b^ ^]^ These clinical and in vivo findings both highlight the distinct role specific point mutations play in disease severity in XLRS. Our observations from the retinas of *Rs1* ^emR209C^ knock‐in mice showed that the causative p.R209C mutation disrupts the octameric structure of the RS1 protein, leading to the accumulation of misfolded RS1 protein. This, in turn, results in the chronic activation of eIF2α pathways, contributing to XLRS pathologies (Figure 4). Remarkably, the combination of salubrinal and AAV8‐based *RS1* delivery significantly reduced both CHOP levels and the number of apoptotic cells, demonstrating a synergistic effect in mitigating retinoschisis and improving impaired ERG responses (Figure 8). In contrast, AAV8‐based *RS1* delivery did not alter CHOP expression or prevent downstream ER stress‐associated apoptotic cell death triggered by the misfolded RS1 protein. In addition, building on salubrinal's ability to alleviate ER stress and its associated apoptosis, our data suggest that therapeutically targeting the chronic activation of the eIF2α pathway, in combination with AAV‐based gene delivery, offers a novel and effective strategy for treating XLRS. Nevertheless, a limitation of the current study is that it focuses solely on XLRS models harboring p.R209C mutations. Further investigation is warranted to explore chronic ER stress‐induced apoptosis and uncover additional biomolecular pathways involved in the pathogenesis associated with other *RS1* point mutations in XLRS.

In conclusion, our findings demonstrate that utilizing two disease models with patient‐specific gene mutations, combined with the identification of novel transcriptional signatures through multiomics approaches, can deepen our understanding of disease mechanisms and advance the development of treatment strategies for XLRS. Furthermore, future applications of a personalized medicine‐based multimodal transcriptomic approach will not only enable precise decoding of the molecular pathogenesis of unexplored ophthalmic degenerations but also aid in discovering new precision‐medicine‐based therapeutic strategies for severe, incurable hereditary diseases like XLRS.

## 4 Experimental Section

### Generation of XLRS‐Like Genetically Engineered Mice Carrying RS1 Mutations at c.625T (p.R209C)

The Transgenic Mouse Model Core Facility of the National Core Facility for Biopharmaceuticals, Ministry of Science and Technology, Taiwan, and the Gene Knockout Core Laboratory of National Taiwan University Centers of Genomic and Precision Medicine provided all the necessary techniques for the production of *Rs1* ^emR209C^ mice. The generation of *Rs1* ^emR209C^ mice with mutation of the specific nucleotide in the *Rs1* gene involved the utilization of CRISPR/Cas9 technology. The detailed procedures for *Rs1* ^emR209C^ mouse generation and other experimental procedures in this work are described in the Supporting Information.

### Statistical Analysis

The data are presented as the mean ± SEM and were analyzed using Microsoft Excel. The statistical analysis was performed using Student's t‐test or one‐way ANOVA. A difference was considered significant when the p‐value was <0.05.

### Ethical Approval

Human tissue samples were collected with informed consent in accordance with the International Ethical Guidelines for Biomedical Research Involving Human Subjects. All experimental procedures and protocols involving these samples were conducted in compliance with the Declaration of Helsinki and were approved by the Institutional Review Board of Taipei Veterans General Hospital (Protocol No. 2022‐08‐003CE).

All animal studies were approved by the Institutional Animal Care and Use Committee (IACUC) of Taipei Veterans General Hospital and were conducted in accordance with IACUC guidelines (Protocol No. 2021–204).

## Conflict of Interest

The authors declare no conflict of interest.

## Author Contributions

Y.C., Y.‐R.W., and C.‐Y.C. contributed equally as first authors to this work. Y.C. integrated all part of this study and managed the project execution. Y.C. and C.‐Y.C. wrote the original manuscript with the input from all co‐authors. Y.C. and C.‐Y.C. provided critical English editing. Y.‐R.W. performed all experiments delineating the *Rs1* ^emR209C^ mouse phenotypes, the eIF2 signaling in *Rs1* ^emR209C^ retina, and the treatment effect of pharmacological eIF2 inhibition. C.‐Y.C. performed spatial transcriptomics (Xenium and Cytassist Visium), single cell RNA‐sequencing. B.‐X.W., and L.‐J.C. conducted the bioinformatics analysis. Y.‐P.Y. assisted the culture of iPSC‐derived retinal organoids and other laboratory works. S.‐Y.C., W.‐C.L., and I.‐C.W. provided valuable technical support. T.‐C.L. provided critical supervision for *Rs1* ^emR209C^ mouse phenotyping. S.‐J.C. and S.‐H.C. supervised the execution of this study. S.‐H.C. was responsible for funding acquisition. I.‐H.C. and W.‐C.C. performed sample preparation and liquid LC/MS proteomics analysis. P.S. performed the pathological studies, including retinal collection and immunofluorescence staining.

## Supporting information



Supporting Information

## Data Availability

The data that support the findings of this study are available in the supplementary material of this article.
