# Supplementary material for: Identifying Multiomic Signatures of X‐Linked Retinoschisis‐Derived Retinal Organoids and Mice Harboring Patient‐Specific Mutation Using Spatiotemporal Single‐Cell Transcriptomics
Source: Adv Sci (Weinh). 2024 Nov 6;12(1):2405818. doi: 10.1002/advs.202405818 (PMC11714187; doi:10.1002/advs.202405818)
Supplement: Supplementary file 1 — Supporting Information [file ADVS-12-2405818-s001.pdf]

ADVANCED SCIENCE
Open Access

Supporting Information

for *Adv. Sci.*, DOI 10.1002/advs.202405818

Identifying Multiomic Signatures of X-Linked Retinoschisis-Derived Retinal Organoids and Mice Harboring Patient-Specific Mutation Using Spatiotemporal Single-Cell Transcriptomics

*Yueh Chien, You-Ren Wu, Chih-Ying Chen, Yi-Ping Yang, Lo-Jei Ching, Bo-Xuan Wang, Wei-Chao Chang, I-Hsun Chiang, Pong Su, Shih-Yu Chen, Wen-Chang Lin, I-Chieh Wang, Tai-Chi Lin, Shih-Jen Chen and Shih-Hwa Chiou**

SUPPLEMENTARY INFORMATION

SUPPLEMENTARY RESULTS:

ScRNA-seq profiling of patient-derived retinal organoids and *Rs1*^{emR209C} retinas

In scRNA-seq, uniform manifold approximation and projection (UMAP) plots were generated for visualization. By analyzing retinas from *Rs1*^{emR209C} and wild-type mice, we identified 11 different clustered cell types, including rod bipolar cells, cone bipolar cells, cones, rods, and Müller glia, among a total of 37,342 cells and 21,014 genes (**Figure 2D Upper; Figure S2A left and S2B Upper; please refer to the Supplementary Information)**. Of these, 19,835 cells (53%) were from *Rs1*^{emR209C} mouse retinas, and 17,507 cells (47%) were from wild-type mouse retinas. The 11 different retina cell types were clustered based on the expression of specific cell markers. For example, rod photoreceptors were categorized by the expression of cyclic nucleotide gated channel subunit alpha 1 (Cnga1). Cone photoreceptors were distinguished based on the expression of phosphodiesterase 6H (Pde6h). RPEs were marked based on the expression of prostaglandin D2 synthase (Ptgds). GABAergic ACs were identified based on the expression of glutamate decarboxylase 1 (Gad1). Glycinergic ACs were identified with the marker gene, solute carrier family 6 member 9 (Slc6a9). Horizontal cells expressed LIM homeobox 1 (Lhx1). Rod bipolar cells expressed the carbonic anhydrase 8 (Car8). Secretagogin (SCGN) was expressed in cone bipolar cells. Claudin 5 (Cldn5) was recognized in endothelial cells. The cell group corresponding to microglia was identified based on the expression of cathepsin S (Ctss). Glutamate-ammonia ligase (Glul) labeled Müller glia (**Figure 2D Upper; Figure S2A left and S2B Upper; please refer to the Supplementary Information**).

For the retinal organoids derived from patient and control iPSCs, 10 different cell types, including rods, cones, and bipolar cells, were classified among a total of 42,293 cells and 30,579 genes (**Figure 2D Lower; Figure S2A right and S2B Lower; please refer to the Supplementary Information**). Rod photoreceptors expressed nuclear receptor subfamily 2 group E member 3 (Nr2e3). Cone photoreceptors were distinguished based on the expression of potassium voltage-gated channel subfamily B member 2 (Kcnb2). Rod progenitors were identified based on the expression of the nuclear factor I b (Nfib). The cell group corresponding to rod/cone progenitors was identified based on the expression of ADP ribosylation factor 4 (Arf4). Bipolar cells expressed the carbonic anhydrase 10 (Ca10). Retinal progenitor cells were marked based on the expression of the marker Secreted Frizzled Related Protein 2 (Sfrp2). Protein kinase CGMP-dependent 1 (Prkg1) labeled Müller glia. Retinal ganglion cells expressed the growth-associated protein 43 (Gap43). Amacrine cells were identified based on the expression of neuregulin 3 (Nrg3). Horizontal cells expressed one-cut

homeobox 1 (Onecut1) (**Figure 2D Lower; Figure S2A right and S2B Lower; please refer to the Supplementary Information**).

SUPPLEMENTARY MATERIALS AND METHODS

Generation of XLRS-like genetically engineered mice carrying RS1 mutations at c.625T (p.R209C)

The Transgenic Mouse Model Core Facility of the National Core Facility for Biopharmaceuticals, Ministry of Science and Technology, Taiwan, and the Gene Knockout Core Laboratory of National Taiwan University Centers of Genomic and Precision Medicine provided all the necessary techniques for the production of *Rs1*^{emR209C} mice. The generation of these mice involved the utilization of CRISPR/Cas9 technology. The generation of *Rs1*^{emR209C} mice with mutation of the specific nucleotide in the *Rs1* gene involved the utilization of CRISPR/Cas9 technology. For *Rs1*^{emR209C} mice, the sgRNA was designed to guide Cas9 cleavage in a targeted sequence of exon 6 of the *Rs1* gene. The mutation changed the amino acid arginine (codon CGA) at #209 to cysteine (codon TGT) in the *Rs1* gene. Selection of the sgRNA sequences following the online resources, including the sgRNA Designer: CRISPRko [1] and Cas-OFFinder [2]. The used sgRNAs were < 3 mismatches and < 25 off-target sites. The sgRNA target sequence with PAM sites (NGG) was 5'- GGCATGTCCGAATTGCCATC -3'. The single-stranded ODN sequence was 5'-TCCCCCCAAAGCTCTCCCTGCAAGTGACAGAACTGAGCTGAAATAGACATCAGGCACACTTGCTGGCACACTCAAGCAGCTCCATTCGAATGGCAATACAGACATGCCAGCCTAGAGGGATCAGTCG -3' and was produced by Integrated DNA Technologies (IDT). Preparation of sgRNA and Cas9 RNA for electroporation followed the commercial protocol, AmpliCap-MaxTM T7 High Yield Message Maker kit (CELLSCRIPT® C-ACM04037). Electroporation was performed on fertilized eggs from C57BL/6J mice. After *in vitro* fertilization, the zygotes (1-cell stage) were subjected to electroporation with Cas9, single guide RNA (sgRNA), and ssODN. The zygotes (2-cell stage) were subsequently transferred to the foster mother, after which the founder mice were obtained. The founder mice were backcrossed to C57BL/6 mice for three generations to minimize off-target CRISPR/Cas9 gene editing changes in each line. Gene sequences of founder mice were validated by PCR, TA cloning, and sequencing. The PCR conditions for genotyping were 95°C for 5 min, followed by 40 cycles of 95°C for 30 sec, 58°C for 30 sec and 72°C for 30 sec, and a final extension at 72°C for 7 min. For *Rs1*^{emR209C} mice, the PCR primer sequences were 5'-GACTAGGCTTCCTCTTTCTCTTT -3'; 5'- AGATATAGCCCCATTCATCCC -3'. Protocol of TA-cloning followed by the T3 Cloning kit (ZGene Biotech Inc., #CT301-02). Finally, 4 male and 2 female founders for *Rs1*^{emR209C} mice were selected. To minimize the off-target effect induced by CRISPR/Cas9 technology, the selected founders were individually backcrossed to C57BL/6 wild-type mice for 3 generations and then intercrossed to obtain homozygous knock-in mice for further experiments.

Finally, mice with a knock-in patient-specific RS1 mutation were generated. **(Figure S1E-S1G)**

Maintenance and differentiation of human iPSCs into retinal organoids

Human induced pluripotent stem cells (hiPSCs) were maintained on Geltrex-coated dishes in StemFlex medium (Gibco), adhering to the manufacturer's instructions. The cells were regularly passaged every 5-7 days upon reaching an approximate confluence of 80%. For the generation of retinal organoids, a previously established method was utilized with minor modifications [3]. In a nutshell, hiPSCs were detached using Vercene (Gibco) and converted into single cells. These cells were then cultured in a low attachment dish, along with a mixture of StemFlex and neural induction medium (NIM), supplemented with 10 μM ROCK inhibitor Y27632 (Sigma). The NIM consisted of DMEM/F12, 1% N2 supplement, 1 × NEAAs, and 2 μg/ml heparin (Sigma). This culture setup initiated the formation of embryonic bodies on Day 0. Gradual transition to NIM was achieved by adjusting the medium ratio to mTeSR1/NIM on Day 3 and Day 5. On Day 7, the embryonic bodies (EBs) were transferred to six-well plates with NIM supplemented with 10% FBS. By Day 16, the medium was switched to retinal differentiation medium (RDM) consisting of DMEM/F12, 2% B27 supplement without vitamin A, 1 × NEAAs, and penicillin/streptomycin. From Days 18 to 25, the central portions of neural clusters were manually separated and further cultured in suspension with RDM, allowing the formation of three-dimensional optic cup structures. Around Days 30 to 40, the retinal organoids were manually isolated. For long-term suspension culture, the medium was supplemented with 10% fetal bovine serum, 100 mM Taurine, and 2 mM GlutaMAX, starting from Day 35. From Days 60 to 90, the culture medium was supplemented with 1 μM retinoic acid to facilitate photoreceptor maturation. After Day 90, N2 supplement replaced B27 in the medium. The cell culture medium was regularly changed every 2-3 days until the desired developmental stage was achieved.

Sanger sequencing

Mutational detection of the *Rs1* gene was performed following a previously described protocol with slight modifications [3a]. Genomic DNA was isolated from the tail of wild-type and $Rs1^{emR209C}$ mice. The primer pairs used for screening the mutation site of the *Rs1* gene were designed as follows: 5’-GACTAGGCTTCCTCTTTCTCTTT-3’ and 5’-AAATCCTTATTGGCATTGAATCC-3’. The size of the PCR products was 268 bp. The sequencing products were analyzed on an ABI PRISM 3700 Genetic Analyzer (Applied Biosystems).

Optical coherence tomography (OCT)

The MICRON IV imaging system (Phoenix-Micron) was utilized for OCT imaging to monitor retinal structural changes and cavity formation. Prior to imaging, mice were subjected to overnight dark adaptation and then anesthetized using 2% to 4% isoflurane inhalation. Topical tropicamide and phenylephrine were administered to achieve pupil dilation. Rectangular volume scans were performed, collecting a total of 100 B-scans. These scans covered an area centered on the optic nerve (ON) head. Additionally, two linear B-scans, averaging 30 frames each, were acquired from the nasal to temporal pole through the ON head.

Electroretinogram (ERG)

Full-field ERGs were performed on male *Rs1*^{emR209C} and wild-type mice to assess retinal function. The experiments were conducted using a Celeris system (Diagnosys). Mice were dark-adapted overnight and anesthetized by inhalation of 2% to 4% isoflurane. Pupil dilation was achieved with topical tropicamide and phenylephrine. Gold loop or wire electrodes were placed on the cornea. For the differential electrode, a gold wire was employed, and the tail was equipped with a connected ground wire. In the dark-adapted ERG, flash intensities ranging from -2 to 2 log cd.s/m^2 were used. Responses were frequency filtered, and a-waves and b-waves were measured. The b-wave-to-a-wave ratio (b/a) was calculated to assess the effects on the isolated postsynaptic response. The ERG signals were amplified, filtered, and analyzed using Espion software (Diagnosys). Throughout the recording sessions, the body temperature was monitored, and lubricating eye gel was administered to prevent corneal dryness.

ScRNAseq library preparation and sequencing

Retinas of wild-type and *Rs1*^{emR209C} mice at 3 weeks, 6 months and 12 months were harvested for scRNAseq. After dissection, the retinas were initially immersed in Hanks' balanced salt solution (HBSS, Sigma), followed by dissociation of retinal cells using the MACS Neural Tissue Dissociation Kit for postnatal neurons (Miltenyi Biotec, 130-094-802) as per the manufacturer's instructions. Subsequently, the dissociated cells were filtered through a 40 μm nylon cell strainer, and washed with washing buffer (1X HBSS, 0.04% BSA). In each sample, a total of 16,000 retinal single cells (at a density of 1000 cells/μm) were resuspended in a washing buffer and prepared for scRNAseq library construction. The library construction for scRNAseq was carried out using the Chromium Next GEM Single Cell 3' Kit v3.1 on a Chromium Connect instrument (10x Genomics). Subsequently, the libraries were sequenced on an Illumina NovaSeq 6000 platform with a target of 20,000 reads per cell.

ScRNAseq data processing

The demultiplexing of sequences from each individual Illumina sequencing dataset was carried out using bcl2fastq v2.20.0.422 (Illumina). Subsequently, the processed sequencing reads were analyzed using 10X Genomics Cell Ranger version 6.0 *[4]*, with the reads aligned to the mouse reference genome mm10 version 3.0.0. To ensure data quality, various steps such as QC filtering, clustering, dimensionality reduction, visualization, and differential gene expression analysis were performed using Seurat v4.04 with R v4.1.0 [5]. Cells were filtered out based on the following criteria: (1) exclusion of doublets, (2) exclusion of cells expressing 15% or more mitochondrial genes, (3) exclusion of cells with fewer than 200 expressed genes (considered as droplets or cellular debris), and (4) exclusion of cells expressing 7,500 or more genes. Each dataset was then subjected to log-normalization using Seurat's NormalizeData function with default parameters. Using Seurat's FindVariableFeature function, we identified 2,000 features in the dataset that displayed significant cell-to-cell variation. Subsequently, we applied linear regression against the number of reads to scale the data using Seurat's ScaleData function with default parameters. The variable genes were then projected onto a lower-dimensional space using principal component analysis (PCA) through Seurat's RunPCA function with default parameters. The number of principal components was determined by examining the plot of variance explained, and a value of 30 principal components (Npcs = 30) was selected. Datasets were integrated using the Seurat integrated default workflow. A shared nearest neighbor graph was constructed based on the Euclidean distance in the low-dimensional subspace using Seurat's FindNeighbors with dims = 1:30 and default parameters. Integrated datasets then underwent nonlinear dimensional reduction and visualization using UMAP. Clusters were identified using a resolution of 0.5 and the Leiden algorithm for the integrated datasets. Cell types were assigned to each cell based on their highest cell type module score created from wild-type controls.

Differential expression analysis and functional enrichment

Differential expression analysis was performed using Seurat's FindMarker function to identify DEGs between *Rs1*^{emR209C} and wild-type groups and between different samples and sample groups within each cluster. DEGs were selected based on a significance threshold of p-value < 0.05. Pathway enrichment analysis was conducted using Ingenuity Pathway Analysis (IPA) to further understand the biological significance of DEGs. This involved annotating gene lists with functional categories and pathways and examining enriched processes in each cluster. The strength of associations was represented by -logFC. Pathways with an adjusted p-value of <0.05

were considered significant. Moreover, to investigate the potential functions and interactions of the DEGs, GO, GSVA, and IPA were performed.

Bulk RNA sequencing and analysis

To extract RNA from retinas of wild-type and Rs1emR209C mice at 3 weeks, 6 months, and 12 months of age, TRIzol (Invitrogen) was used following the provided instructions. The purified RNA was employed for constructing sequencing libraries using the TruSeq Stranded mRNA Library Prep Kit (Illumina, San Diego, CA, USA) according to the manufacturer's guidelines. Initially, mRNA was isolated from 1 μg of total RNA using oligo(dT)-coupled magnetic beads and fragmented at an elevated temperature. Subsequently, first-strand cDNA was synthesized using reverse transcriptase and random primers. Double-strand cDNA was generated, and DNA fragments underwent adenylation at the 3' ends before ligating adaptors. The resulting products were enriched by PCR and purified using the AMPure XP system (Beckman Coulter, Beverly, USA). The quality of the libraries was assessed using the Qsep400 System (Bioptic Inc., Taiwan), and their quantity was determined with the Qubit 2.0 Fluorometer (Thermo Scientific, Waltham, MA, USA). The qualified libraries were then sequenced on an Illumina NovaSeq 6000 platform, generating 150 bp paired-end reads. The original image data were converted into sequence data through base calling, resulting in raw data or raw reads saved as FASTQ files. All FASTQ sequences were aligned to the mouse genome mm9 (GRCm37). For the alignment and quantification steps, Kallisto [6] was used for file format conversion. DESeq2 (version 1.22.2) was employed for differential expression gene analysis. Genes exhibiting a 1.5-fold expression over the background with a q-value (FDR) less than 0.05 were considered as differentially expressed genes (DEGs) in each cluster.

Spatial transcriptomics

Xenium In Situ

The custom gene panel for retinas was designed based on the scRNA-seq data. Xenium In Situ Gene Expression technology was performed using the Xenium in Situ Gene Expression Reagent Kits (10x Genomics) following the manufacturer's instructions. Briefly, formalin-fixed, paraffin-embedded mice retina tissue sections (5 μm) were cut and placed on the Xenium Slides (10x Genomics, PN-3000941). After probe hybridization, ligation, and amplification, we immediately loaded the Xenium slide onto the Xenium Analyzer. Post Xenium, retina sections were stained with hematoxylin and eosin to obtain the histological data that can be combined with gene expression data from the same tissue section. Gene expression data was analyzed using Xenium Explorer software. Initially, we clustered the known gene sets, identifying

corresponding cells and their cell types. Subsequently, we conducted a more in-depth analysis of the target pathogenic pathway. Each transcript was transformed into a heatmap, with darker colors indicating a higher transcript count. Comparisons were made across different samples.

Cytassist Visium

The 5 μm sections of paraffin-embedded retinas from both wild-type and Rs1^{emR209C} samples underwent deparaffinization, H&E staining, and imaging using a Nikon Eclipse T2 microscope, following the 10x Genomics protocol (CG000520). For the construction of the sequencing library, the Visium CytAssist FFPE Spatial Gene Expression 6.5 mm (Mouse; 10x Genomics) and the Visium CytAssist instrument (10x Genomics) were utilized. Subsequently, the libraries were sequenced on an Illumina NovaSeq 6000 platform. After library construction and sequencing, the Tissue Microarray (TMA) 'count' method in Space Ranger (v2.1.0; 10x Genomics) was employed to align the probe reads to the mouse reference genome (mm10) using a short-read probe alignment algorithm. The resulting count matrix, along with the accompanying H&E images, were further analyzed using the R package Seurat (v.4.3.0). The gene-count matrices were normalized using SCTransform and integrated into a single object for joint processing. Integration of datasets was performed using Seurat's default workflow for integration. A shared nearest neighbor graph was constructed based on the top 30 principal components (PCs) and default conditions using Seurat's FindNeighbors function, utilizing the Euclidean distance in the low-dimensional subspace. The expression levels of specific genes were visualized using the SpatialDimPlot and SpatialFeaturePlot functions. Differential expression gene analysis was conducted using the FindMarkers function.

Immunohistochemistry

Immunohistochemistry staining was performed as previously described [7]. The floating retinal organoids were collected, fixed with 4% paraformaldehyde for 30 min, paraffin-embedded, and sectioned. The mouse retinas were collected, fixed with Hartmann’s fixative, paraffin-embedded, and sectioned. Paraffin-embedded retinal organoids and retinas were stained with H&E. For IF, mouse eyeballs were embedded in Tissue-Tek O.C.T. Compound (Sakura), and frozen sectioned using a cryostat (Leica). After blocking and permeating steps, the cells were incubated with primary antibodies and secondary antibodies. Images were acquired with an Olympus FV3000 laser scanning confocal microscope.

TUNEL staining

To detect retinal cell apoptosis, the TUNEL (terminal deoxynucleotidyl transferase dUTP nick-end labeling) In Situ Apoptosis Kit from Elabscience Biotechnology was employed, following the manufacturer's instructions. Initially, each section was treated with proteinase K and incubated at 37°C for 10 minutes. Subsequently, TdT labeling solution was applied to each section and incubated at 37°C for 30 minutes. The slides were then washed with PBS three times, with each wash lasting 5 minutes. In addition, frozen retina sections were stained with 4',6'-diamino-2-phenylindole (DAPI) to facilitate the visualization of the cell nucleus.

Western blot

Mouse retinas were lysed using RIPA lysis buffer (Millipore) containing a protease inhibitor cocktail (Roche). Protein concentration normalization was achieved by utilizing Protein Assay Dye Reagent Concentrate from Bio-Rad. Equal amounts of proteins were denatured in either a 10% (reducing conditions) or 6% (nonreducing conditions) Bis-Tris precast polyacrylamide gel, along with an SDS mixture (10 mM Tris, pH 6.8, 1% SDS, 10% glycerol), with or without 4% β-mercaptoethanol. The gel was then transferred onto a membrane using transfer buffer (25 mM Tris, 192 mM glycine, and 20% methanol) for a duration of 2 hours. After blocking with 5% skim milk in TBST for 1 hour, the membranes were incubated overnight at 4°C with primary antibodies targeting RS1 at a dilution of 1:1000. The membranes were blocked in 5% skim milk in TBST for 1 hour and incubated overnight at 4°C with primary antibodies against RS1 (1:1000 dilution), p-EIF2A, EIF2A, ATF4, BiP, CHOP, GAPDH, and β-actin (all at a 1:20000 dilution). Following three washes with TBST, the membranes were incubated with HRP-conjugated secondary antibodies (1:1000 dilution in 5% BSA) for 60 minutes at room temperature. Subsequently, the membranes were washed three times with TBST, detected using Immobilon ECL Ultra Western HRP Substrate (Millipore), and imaged using UVP ChemStudio PLUS (Analytik Jena).

Salubrinal treatment

Rs1$^{\mathrm{emR209C}}$ mice were intraperitoneally injected once daily starting at 3 weeks of age with 1 mg/kg salubrinal (Sigma) at a volume of 100 μL for one month. The vehicle group received an equal volume of phosphate-buffered saline (pH 7.2). Mice were monitored daily for any adverse effects.

AAV-RS1 delivery

The design of AAV-RS1 vector and the procedure to deliver AAV-RS1 were conducted based on a previously described protocol with modifications [8]. Rs1^{emR209C} mice at 3-week-old age were subjected to the intravitreal injection of 2×10^9 viral vector

genomes per eye using a Hamilton syringe equipped with a 33-gauge needle. Prior to injection, animals were anesthetized with 2% to 4% isoflurane. Retinal structure and electrophysiological functions were assessed a month after the AAV-mediated RS1 injection.

Data Availability

The sequencing datasets of mouse retinas for this project have been deposited in the Gene Expression Omnibus (GEO) database under accession numbers GSE277399, GSE277403, and GSE277406.

TABLE S1. qRT-PCR primers

	**Forward primer**	**Reverse primer**
**sXBP1**	CTGAGTCCGAATCAGGTGCAG	GTCCATGGGAAGATGTTCTGG
**ATF4**	GTGGCCAAGCACTTGAAACC	GGAAAAGGCATCCTCCTTGC
**HSPA5**	TTCAGCCAATTATCAGCAAACTCT	TTTTCTGATGTATCCTCTTCACCAGT
**DDIT3**	CATACACCACCACACCTGAAAG	CCGTTTCCTAGTTCTTCCTTGC
**DNAJC3**	ACGCCTTTGACGGTGCCGATTA	AAGTCGCTGATGGCTTTCCTGG
**GRP94**	GACCTTCGGGTTCGTCAGAG	AGCCTTCTCGGCTTTTACCC
**β-actin**	TTGCTGACAGGATGCAGAAG	GTACTTGCGCTCAGGAGGAG
**LAMP1**	CAGCACTCTTTGAGGTGAAAAAC	ACGATCTGAGAACCATTCGCA
**LAMP2**	ATATGTGCAACAAAGAGCAGGT	TGCCAATTAGGTAAGCAATCACT
**SOD1**	CAGAAGGCAAGCGGTGAAC	CAGCCTTGTGTATTGTCCCCATA
**Catalase**	GGACGCTCAGCTTTTCATTC	TTGTCCAGAAGAGCCTGGAT
**Caspase 3**	TGCAGCATGCTGAAGCTGTA	GAGCATGGACACAATACACG
**Fas**	GCTGCAGACATGCTGTGGATC	TCACAGCCAGGAGAATCGCAG

TABLE S2. Antibody information

Antibody	Species	Manufacturer	Cat. No.
anti-PKCa	Rabbit	Cell Signaling	2056
anti-DLG4	Mouse	Cell Signaling	36233
anti-GFAP	Rabbit	abcam	ab7260
anti-RS1	Mouse	abcam	ab167579
anti-BiP	Rabbit	abcam	ab21685
anti-EIF2A	Rabbit	Cell Signaling	9722
anti-Phospho-EIF2A (Ser51)	Rabbit	Cell Signaling	9721
anti-ATF4	Rabbit	Cell Signaling	11815
anti-CHOP	Mouse	Cell Signaling	2895
HRP-conjugated anti-GAPDH	Mouse	Proteintech	HRP-60004
anti-Puromycin	Mouse	Millipore	MABE343
anti-PERK	Rabbit	Cell Signaling	3192
anti-phospho-PERK (Thr982)	Rabbit	Affinity Biosciences	DF7576
atnti-GNB3	Rabbit	Proteintech	10081-1-AP
anti-EDN2	Rabbit	NOVUS	NBP1-87942

SUPPLEMENTARY FIGURE LEGENDS

Figure S1. Generation of XLRS patient-specific iPSC-differentiated retinal organoids and *Rs1*^{emR209C} mice. (A) Phenotypic determination of normal and XLRS patient-specific iPSC-differentiated retinal organoids at post-induction 150 days under bright field microscopy and H & E staining. (B) The bright-field images of healthy control and XLRS patient-derived retinal organoids at 90 days, 120 days, and 150 days postinduction. (C) The Sanger Sequencing result of human iPSC control (top) and XLRS human patient iPSC (bottom). (D) Confirmation of *RS1* point mutation site of XLRS patient-specific iPSCs using Sanger sequencing. (E) A graph showing PCR targets and primer sequences to determine wild-type and knock-in allele. (F) A scheme of the breeding setup to generate *Rs1*^{emR209C} mice colony. (G) The result of Sanger Sequencing showed nucleotide changes in the founder *Rs1*^{emR209C} mice. (H) The Sanger Sequencing of wild-type and *Rs1*^{emR209C} mice. (I) Representative images and (J) quantification of DAPI stained photoreceptor cell density in the ONL from *Rs1*^{emR209C} and wild-type retinas at different ages. (K) Quantification of GRAP-positive areas from *Rs1*^{emR209C} and wild-type retinas at different ages (L) Scotopic a-wave, (M) scotopic b-wave, and (N) a-wave implicit time of *Rs1*^{emR209C} and age-matched wild-type mouse retinas at different ages.

Figure S2. DEG identification and functional analysis of human retinal organoids and mouse retinas. (A) Left: The heat map of average expression of each gene in each cell type in mouse retinas. Right: The heat map of average expression of each gene in each cell type in human retinal organoids. (B) Upper: A separate view of the expression of cell-type specific marker genes in each cell type in mouse retinas. Lower: A separate view of the expression of cell-type specific marker genes in each cell type in human retinal organoids. (C) Clustering of different cell types based on cell type-specific markers in XLRS patient-specific *RS1*$^{R209C/y}$ mutation and control iPSC differentiated retinal organoids. (D) Up-regulated and down-regulated DEGs of scRNA-seq dataset in bipolar cells from from XLRS patient iPSC- and ctrl iPSC-derived retinal organoids at different post-induction days. (E) Upper: The volcano plot showing the DEGs in cone and bipolar cells of 3-week-old, 6-month-old and 12-month-old mouse retinas. Lower: The volcano plot showing the DEGs in rod, cone and bipolar cells of human retinal organoids.

(F) The enrichment of eIF2 signaling and eIF2-related signaling pathways in human retinal organoids (Upper) and mouse retinas (Lower).

Figure S3. Visualization of retinal cell types using Xenium analysis. (A) Xenium analysis visualizes selected marker genes associated with specific retinal cell types. (B) Identification of specific retinal cell types using selected marker genes in mouse retinas. (C) Analysis of the eIF2 signaling pathway after completing a layered analysis of the retinal cells from *Rs1*^{emR209C} retinas and wild-type retinas. (D) The functional analysis of overlapping enriched genes in RNAseq, scRNAseq and spatial transcriptome. (E) Heatmap shows the expression of CytAssist Visium-detected enriched genes in bulk RNAseq and the rod, cone, and bipolar cell data from scRNA-seq. CytAssist Visium-captured spots shows the gene expression of *Edn2* and *Gnb3* in wild-type and *Rs1*^{emR209C} retinas.

Figure S4. Comparison of selected gene expression in *Rs1*^{emR209C} and wild-type mouse retinas. qRT-PCR results of the gene expression associated with the ER stress pathway and other disease-related pathways in *Rs1*^{emR209C} and wild-type mouse retinas.

Figure S5. Schematic procedures for salubrinal treatment. Experimental procedures showing the phenotypic examination of salubrinal-treated *Rs1*^{emR209C} mice using OCT imaging, H&E staining, and electroretinogram.

SUPPLEMENTARY REFERENCES

[1] J. G. Doench, N. Fusi, M. Sullender, M. Hegde, E. W. Vaimberg, K. F. Donovan, I. Smith, Z. Tothova, C. Wilen, R. Orchard, H. W. Virgin, J. Listgarten, D. E. Root, *Nat Biotechnol* **2016**, *34* (2), 184, https://doi.org/10.1038/nbt.3437.

[2] S. Bae, J. Park, J. S. Kim, *Bioinformatics* **2014**, *30* (10), 1473, https://doi.org/10.1093/bioinformatics/btu048.

[3] a) K. C. Huang, M. L. Wang, S. J. Chen, J. C. Kuo, W. J. Wang, P. N. Nhi Nguyen, K. J. Wahlin, J. F. Lu, A. A. Tran, M. Shi, Y. Chien, A. A. Yarmishyn, P. H. Tsai, T. C. Yang, W. N. Jane, C. C. Chang, C. H. Peng, T. M. Schlaeger, S. H. Chiou, *Stem Cell Reports* **2019**, *13* (5), 906, https://doi.org/10.1016/j.stemcr.2019.09.010; b) S. K. Ohlemacher, C. L. Iglesias, A. Sridhar, D. M. Gamm, J. S. Meyer, *Curr Protoc Stem Cell Biol* **2015**, *32*, 1H 8 1, https://doi.org/10.1002/9780470151808.sc01h08s32.

[4] G. X. Zheng, J. M. Terry, P. Belgrader, P. Ryvkin, Z. W. Bent, R. Wilson, S. B. Ziraldo, T. D. Wheeler, G. P. McDermott, J. Zhu, M. T. Gregory, J. Shuga, L. Montesclaros, J. G. Underwood, D. A. Masquelier, S. Y. Nishimura, M. Schnall-Levin, P. W. Wyatt, C. M. Hindson, R. Bharadwaj, A. Wong, K. D. Ness, L. W. Beppu, H. J. Deeg, C. McFarland, K. R. Loeb, W. J. Valente, N. G. Ericson, E. A. Stevens, J. P. Radich, T. S. Mikkelsen, B. J. Hindson, J. H. Bielas, *Nat Commun* **2017**, *8*, 14049, https://doi.org/10.1038/ncomms14049.

[5] T. Stuart, A. Butler, P. Hoffman, C. Hafemeister, E. Papalexi, W. M. Mauck, 3rd, Y. Hao, M. Stoeckius, P. Smibert, R. Satija, *Cell* **2019**, *177* (7), 1888, https://doi.org/10.1016/j.cell.2019.05.031.

[6] N. L. Bray, H. Pimentel, P. Melsted, L. Pachter, *Nat Biotechnol* **2016**, *34* (5), 525, https://doi.org/10.1038/nbt.3519.

[7] P. H. Tsai, Y. Chien, M. L. Wang, C. H. Hsu, B. Laurent, S. J. Chou, W. C. Chang, C. S. Chien, H. Y. Li, H. C. Lee, T. I. Huo, J. H. Hung, C. H. Chen, S. H. Chiou, *Nucleic Acids Res* **2019**, *47* (19), 10115, https://doi.org/10.1093/nar/gkz801.

[8] D. Marangoni, R. A. Bush, Y. Zeng, L. L. Wei, L. Ziccardi, C. Vijayasarathy, J. T. Bartoe, K. Palyada, M. Santos, S. Hiriyanna, Z. Wu, P. Colosi, P. A. Sieving, *Mol Ther Methods Clin Dev* **2016**, *5*, 16011, https://doi.org/10.1038/mtm.2016.11.

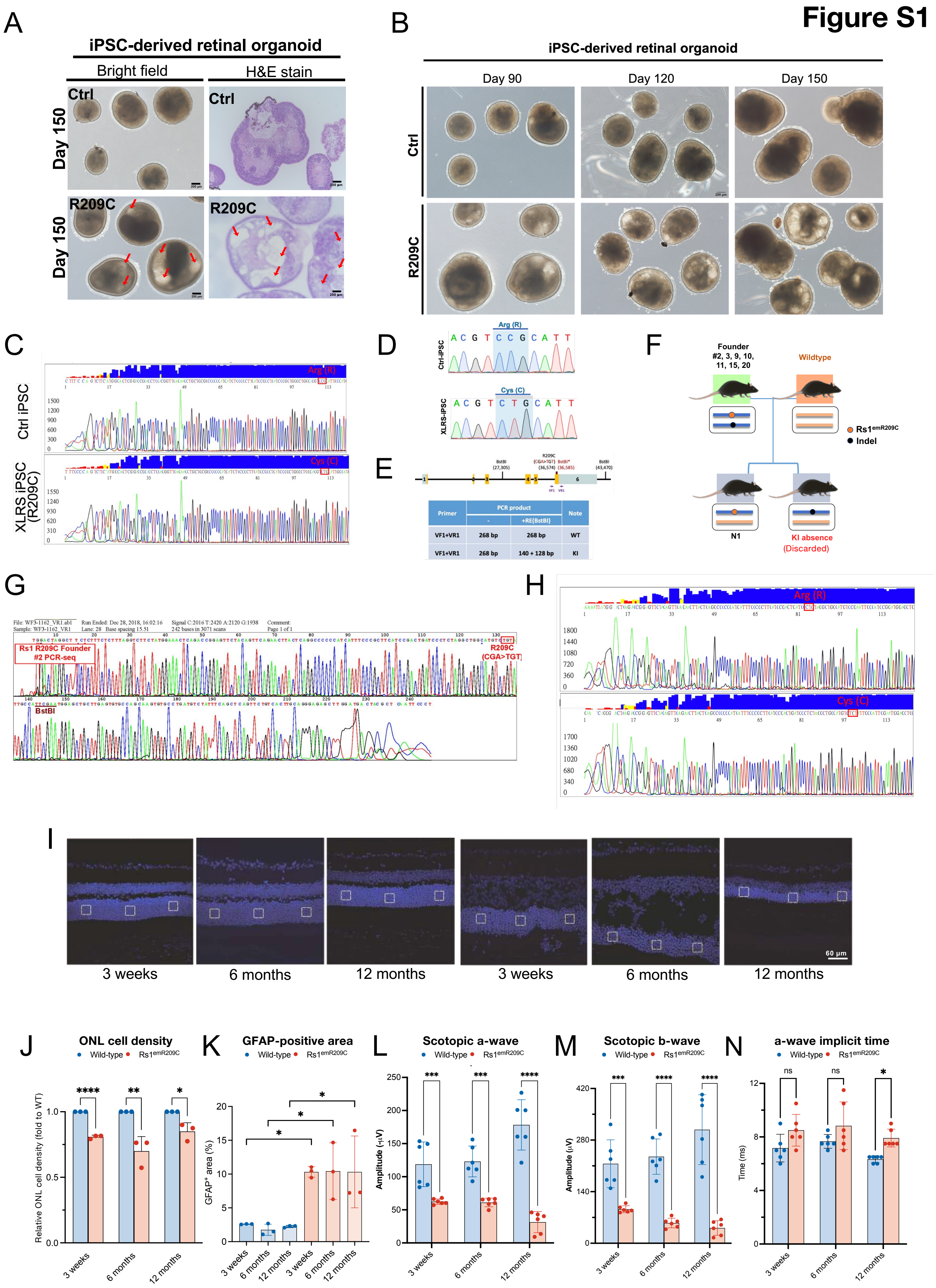
A
iPSC-derived retinal organoid
Bright field
H&E stain
Ctrl
R209C
Day 150
B
iPSC-derived retinal organoid
Day 90
Day 120
Day 150
Ctrl
R209C
C
Ctrl iPSC
XLRS iPSC (R209C)
Arg (R)
Cys (C)
D
Ctrl-iPSC
XLRS-iPSC
Arg (R)
Cys (C)
E
BstBI (27,305)
R209C (CGA>TGT) BstBI* (36,574) (36,585)
BstBI (43,470)
VF1
VR1
Primer
PCR product
-
+RE(BstBI)
Note
VF1+VR1
268 bp
268 bp
WT
VF1+VR1
268 bp
140 + 128 bp
KI
F
Founder #2, 3, 9, 10, 11, 15, 20
Wildtype
Rs1emR209C
Indel
N1
KI absence (Discarded)
G
Rs1 R209C Founder #2 PCR-seq
R209C (CGA>TGT)
BstBI
H
Arg (R)
Cys (C)
I
3 weeks
6 months
12 months
3 weeks
6 months
12 months
60 μm
J
ONL cell density
Wild-type
Rs1emR209C
Relative ONL cell density (fold to WT)
****
**
*
3 weeks
6 months
12 months
K
GFAP-positive area
Wild-type
Rs1emR209C
GFAP+ area (%)
*
*
*
3 weeks
6 months
12 months
3 weeks
6 months
12 months
L
Scotopic a-wave
Wild-type
Rs1emR209C
Amplitude (-μV)
***
***
****
3 weeks
6 months
12 months
M
Scotopic b-wave
Wild-type
Rs1emR209C
Amplitude (μV)
***
****
****
3 weeks
6 months
12 months
N
a-wave implicit time
Wild-type
Rs1emR209C
Time (ms)
ns
ns
*
3 weeks
6 months
12 months

Figure S1

Figure S2

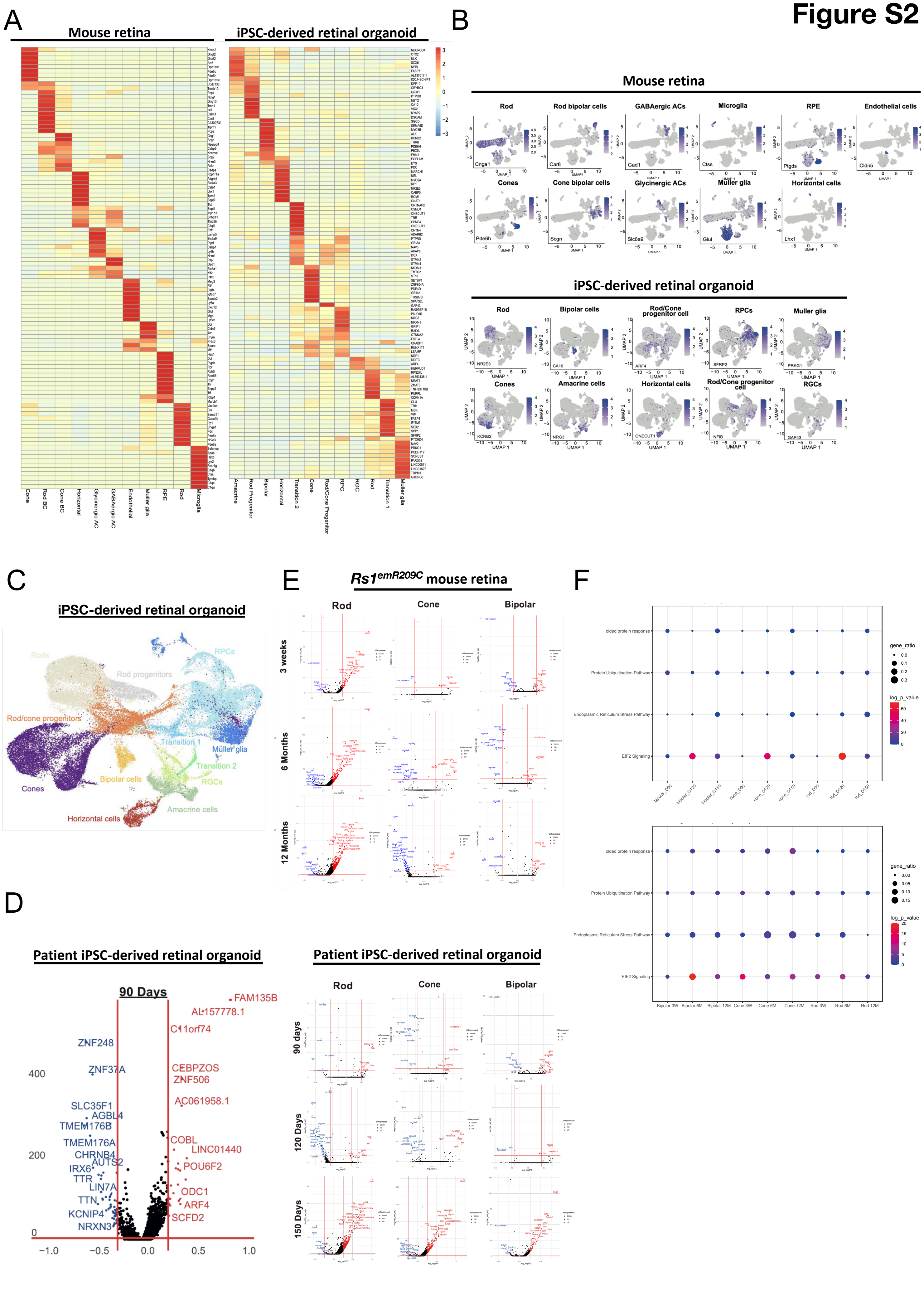
A
Mouse retina
iPSC-derived retinal organoid
Cone
Rod BC
Cone BC
Horizontal
Glycinergic AC
GABAergic AC
Endothelial
Muller glia
RPE
Rod
Microglia
Amacrine
Rod Progenitor
Bipolar
Horizontal
Transition 2
Cone
Rod/Cone Progenitor
RPC
RGC
Rod
Transition 1
Muller glia
B
Mouse retina
Rod
Rod bipolar cells
GABAergic ACs
Microglia
RPE
Endothelial cells
Cones
Cone bipolar cells
Glycinergic ACs
Muller glia
Horizontal cells
iPSC-derived retinal organoid
Rod
Bipolar cells
Rod/Cone progenitor cell
RPCs
Muller glia
Cones
Amacrine cells
Horizontal cells
Rod/Cone progenitor cell
RGCs
C
iPSC-derived retinal organoid
Rods
Rod progenitors
RPCs
Rod/cone progenitors
Transition 1
Müller glia
Transition 2
Bipolar cells
RGCs
Cones
Amacrine cells
Horizontal cells
E
Rs1emR209C mouse retina
Rod
Cone
Bipolar
3 weeks
6 Months
12 Months
F
olded protein response
Protein Ubiquitination Pathway
Endoplasmic Reticulum Stress Pathway
EIF2 Signaling
gene_ratio
log_p_value
bipolar_D90
bipolar_D120
bipolar_D150
cone_D90
cone_D120
cone_D150
rod_D90
rod_D120
rod_D150
Bipolar 3W
Bipolar 6M
Bipolar 12M
Cone 3W
Cone 6M
Cone 12M
Rod 3W
Rod 6M
Rod 12M
D
Patient iPSC-derived retinal organoid
90 Days
FAM135B
AL157778.1
C11orf74
ZNF248
ZNF37A
CEBPZOS
ZNF506
AC061958.1
SLC35F1
AGBL4
TMEM176B
TMEM176A
COBL
CHRNB4
AUTS2
LINC01440
IRX6
POU6F2
TTR
LIN7A
ODC1
TTN
ARF4
KCNIP4
SCFD2
NRXN3
400
200
0
−1.0
−0.5
0.0
0.5
1.0
Patient iPSC-derived retinal organoid
Rod
Cone
Bipolar
90 days
120 Days
150 Days

Figure S3

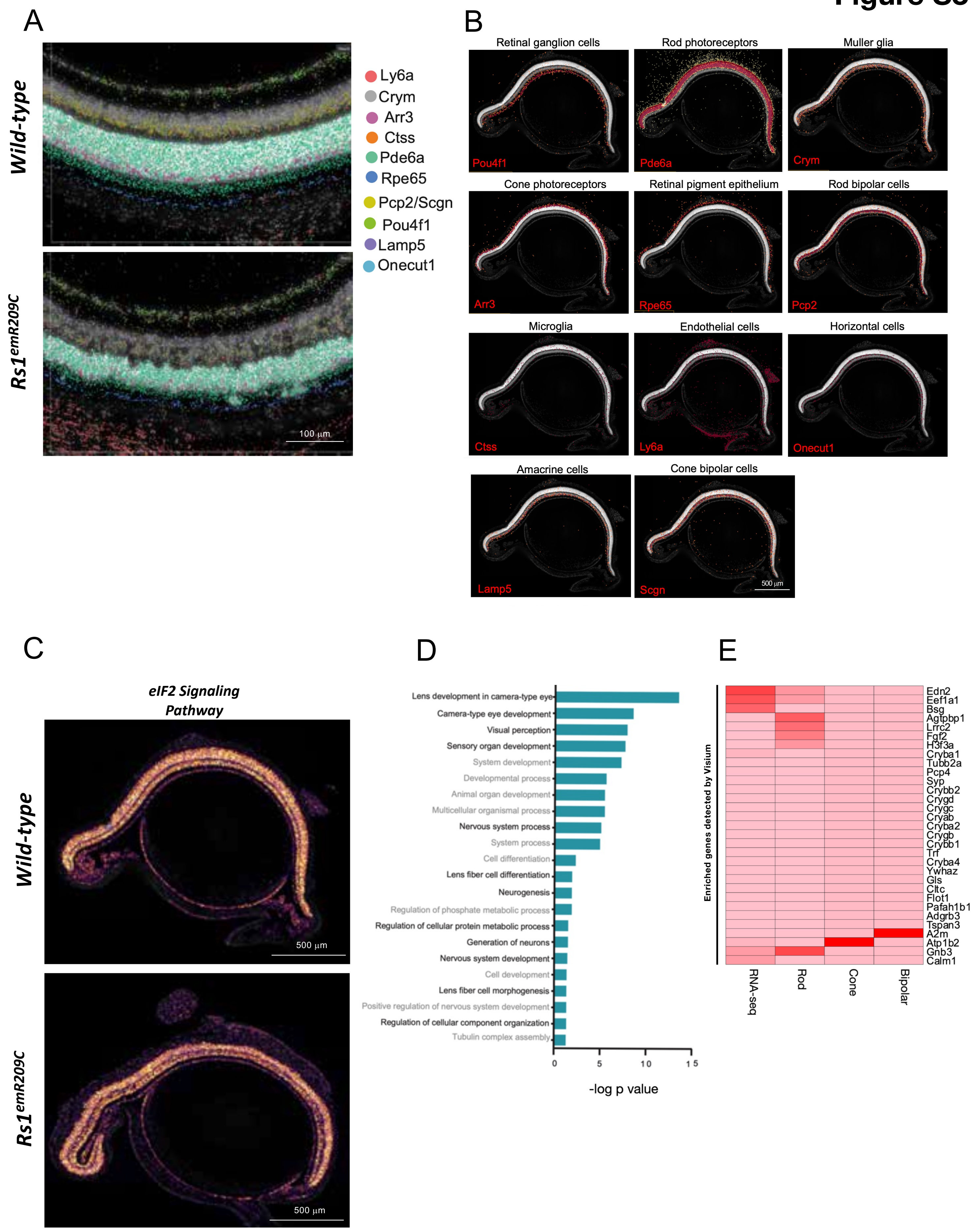

A
Wild-type
Rs1emR209C
Ly6a
Crym
Arr3
Ctss
Pde6a
Rpe65
Pcp2/Scgn
Pou4f1
Lamp5
Onecut1
100 µm
B
Retinal ganglion cells
Pou4f1
Rod photoreceptors
Pde6a
Muller glia
Crym
Cone photoreceptors
Arr3
Retinal pigment epithelium
Rpe65
Rod bipolar cells
Pcp2
Microglia
Ctss
Endothelial cells
Ly6a
Horizontal cells
Onecut1
Amacrine cells
Lamp5
Cone bipolar cells
Scgn
500 µm
C
eIF2 Signaling Pathway
Wild-type
Rs1emR209C
500 µm
D
Lens development in camera-type eye
Camera-type eye development
Visual perception
Sensory organ development
System development
Developmental process
Animal organ development
Multicellular organismal process
Nervous system process
System process
Cell differentiation
Lens fiber cell differentiation
Neurogenesis
Regulation of phosphate metabolic process
Regulation of cellular protein metabolic process
Generation of neurons
Nervous system development
Cell development
Lens fiber cell morphogenesis
Positive regulation of nervous system development
Regulation of cellular component organization
Tubulin complex assembly
0
5
10
15
-log p value
E
Enriched genes detected by Visium
Edn2
Eef1a1
Bsg
Agtpbp1
Lrrc2
Fgf2
H3f3a
Cryba1
Tubb2a
Pcp4
Syp
Crybb2
Crygd
Crygc
Cryab
Cryba2
Crygb
Crybb1
Trf
Cryba4
Ywhaz
Gls
Cltc
Flot1
Pafah1b1
Adgrb3
Tspan3
A2m
Atp1b2
Gnb3
Calm1
RNA-seq
Rod
Cone
Bipolar

Figure S4

A

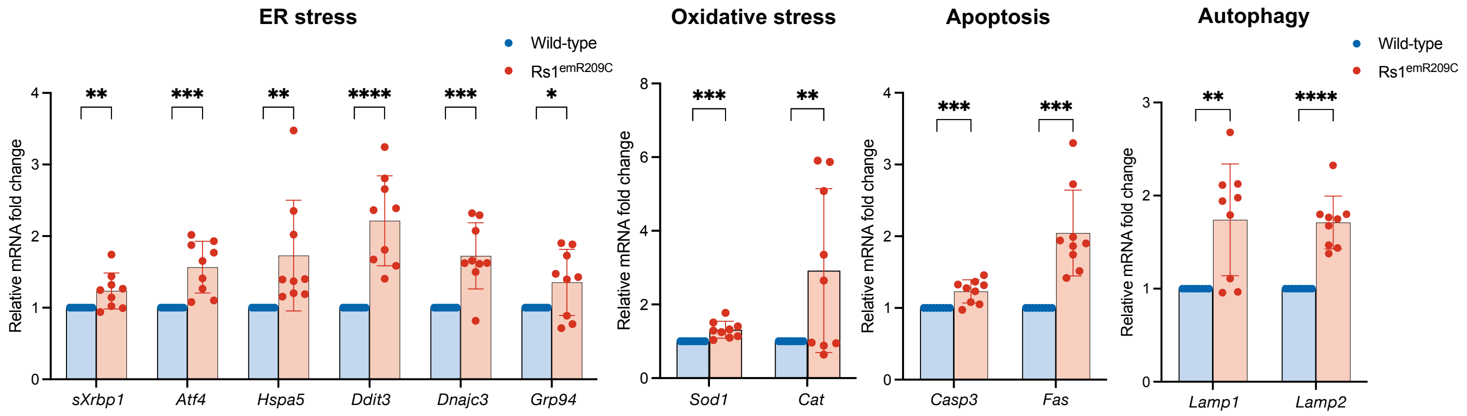

ER stress
Oxidative stress
Apoptosis
Autophagy
Wild-type
Rs1emR209C
Relative mRNA fold change
**
***
****
*
sXrbp1
Atf4
Hspa5
Ddit3
Dnajc3
Grp94
Sod1
Cat
Casp3
Fas
Lamp1
Lamp2

Figure S5

A

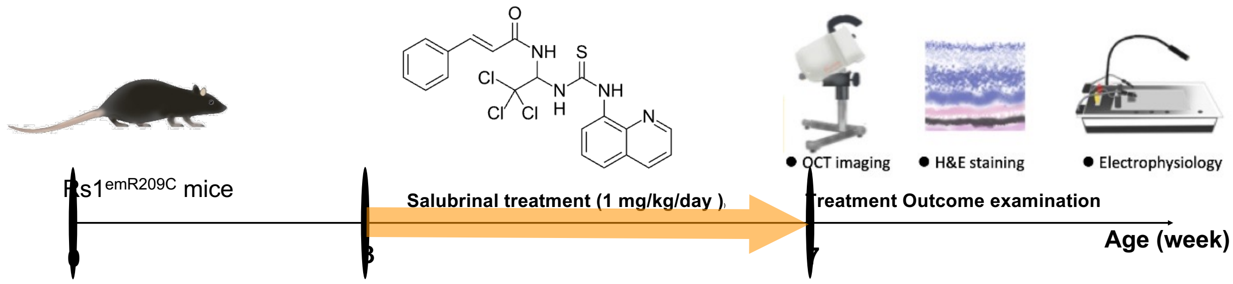

Rs1^{emR209C} mice
O
NH
S
Cl
N
H
NH
Cl
Cl
N
OCT imaging
H&E staining
Electrophysiology
Salubrinal treatment (1 mg/kg/day)
Treatment Outcome examination
Age (week)
